# Supplementary material for: Modeling Glycan Processing Reveals Golgi-Enzyme Homeostasis upon Trafficking Defects and Cellular Differentiation
Source: Cell Rep. 2019 Apr 23;27(4):1231–1243.e6. doi: 10.1016/j.celrep.2019.03.107 (PMC6486481; doi:10.1016/j.celrep.2019.03.107)
Supplement: Document S1. Figures S1–S6 and Tables S1, S2, and S4–S7 [file mmc1.pdf]

Cell Reports, Volume *27*

Supplemental Information

Modeling Glycan Processing Reveals Golgi-Enzyme Homeostasis upon Trafficking Defects and Cellular Differentiation

Peter Fisher, Hannah Spencer, Jane Thomas-Oates, A. Jamie Wood, and Daniel Ungar

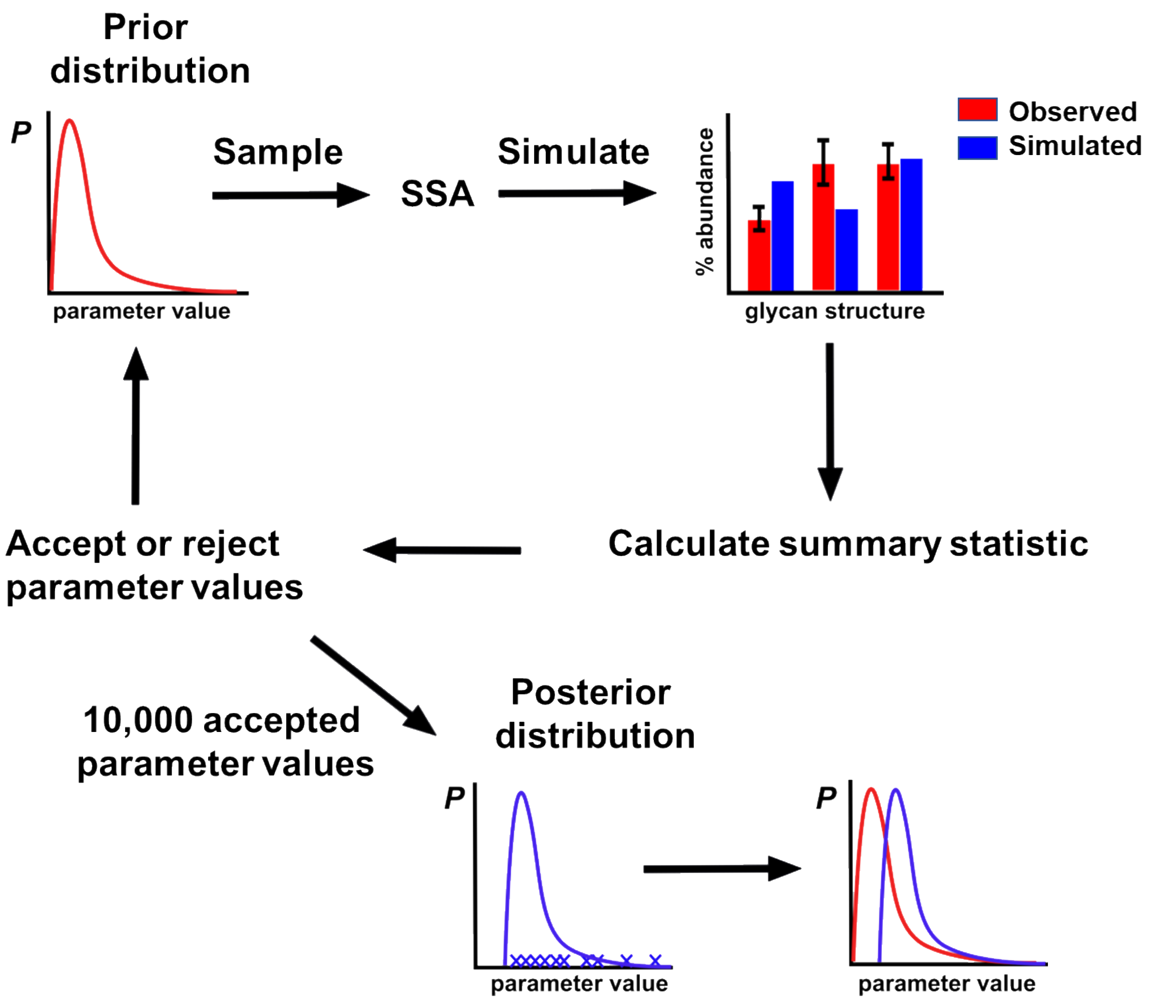

Figure S1. related to figure 1 | Pictorial representation of the Approximate Bayesian Computation fitting procedure.

A parameter value is sampled from prior distribution and used to simulate a glycan profile. The simulated glycan profile is compared to the observed glycan profile and a summary statistic calculated. The parameter value is accepted if the similarity between the simulated and observed glycan profiles is below a user defined threshold, otherwise the parameter value is rejected. This process is repeated until the lowest possible score is achieved at the optimal acceptance ratio, then 10,000 values are sampled in order to construct a posterior distribution.

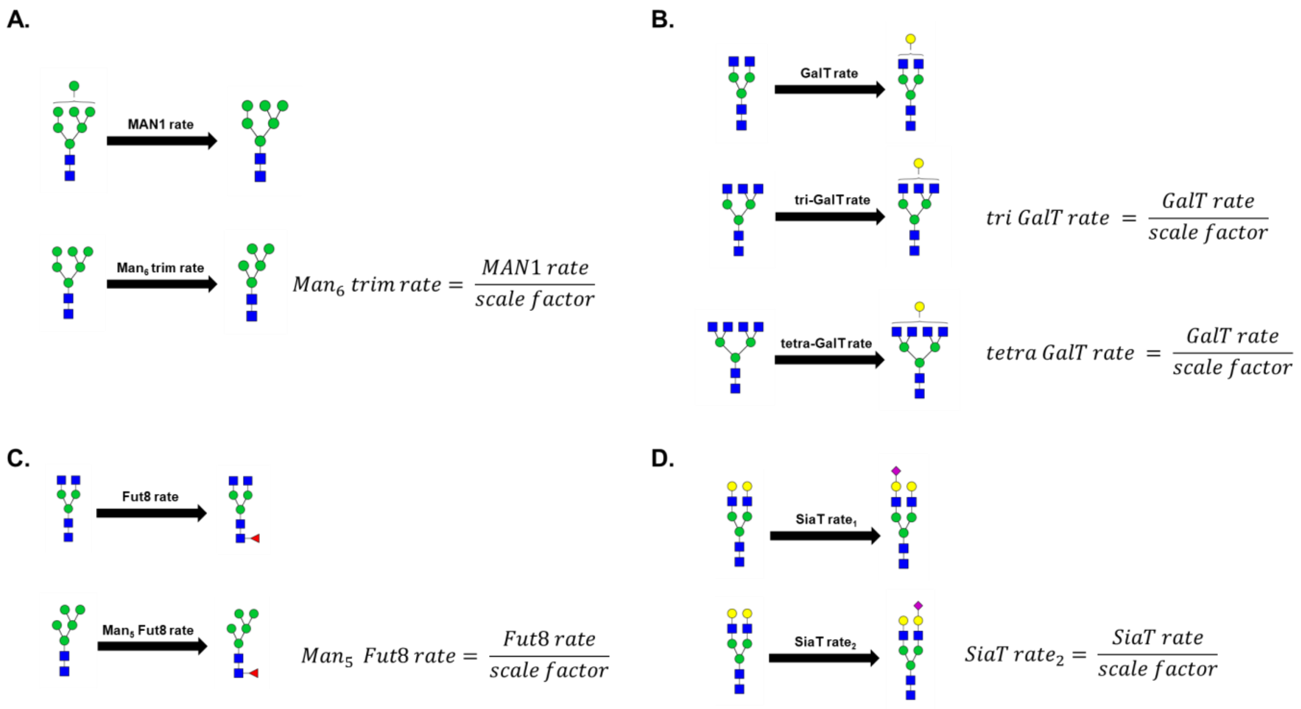

Figure S2. related to figure 2 | Graphical representation of the scale-factors used to simulate substrate specific activities.

(A) For MAN1 to convert $GlcNAc_2Man_6$, (B) For GalT to convert bi-, tri- and tetra-antennary glycans, (C) For FUT8 to convert $GlcNAc_2Man_5$, and (D) For SiaT to add sialic acid to different antennae.

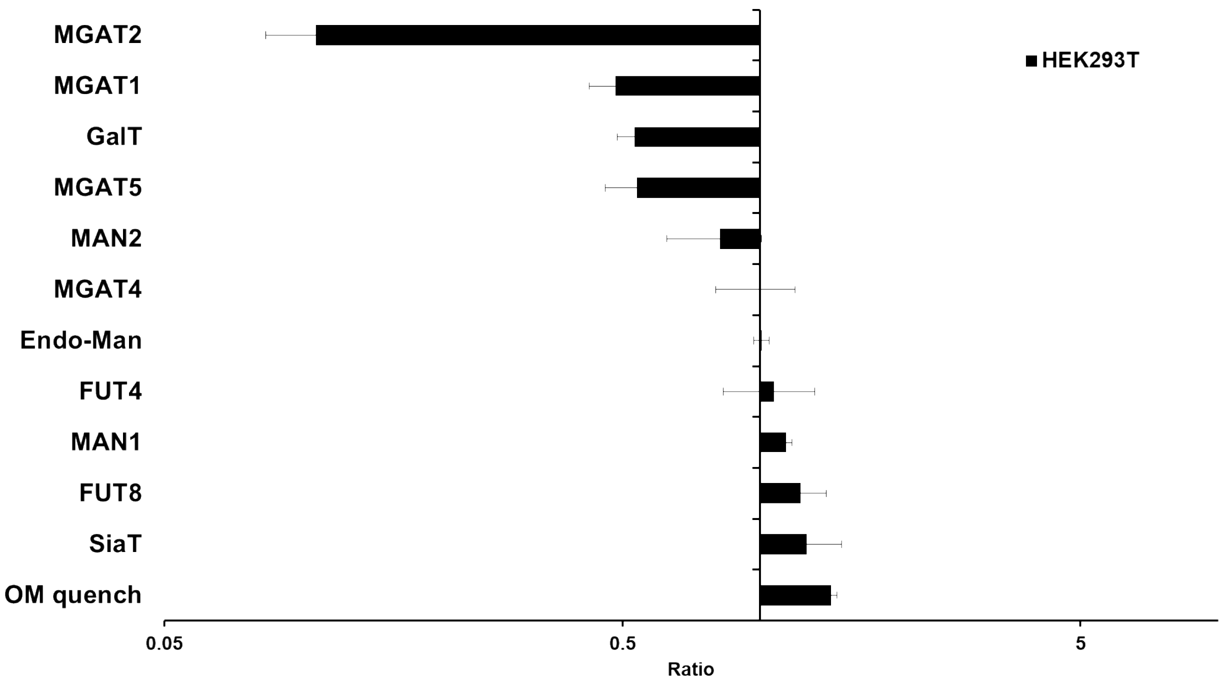

Figure S3. related to figure 4 | Predicted total activity changes between WT HEK293T and Cog4KO HEK293T cells.

This figure shows the graph as displayed in figure 4C, but with MGAT2 included. The reason to exclude MGAT2 from figure 4C is as follows:

Fitting of the WT profile was insensitive to MGAT2 alterations due to the very high activity obtained from the literature during initial parameterization. However, fitting of the Cog4KO profile was sensitive to this parameter. MGAT2 activities were therefore manually reduced in all cisternae prior to Cog4KO fitting, resulting in this enzyme displaying the largest change in activity in this figure. However, the low MGAT2 activities resulting from fitting to the Cog4KO profile did not change the simulated WT glycan profile at all. This implies that the change of this parameter upon Cog4KO is not a biologically meaningful result, but purely due to it being undefined in the WT case.

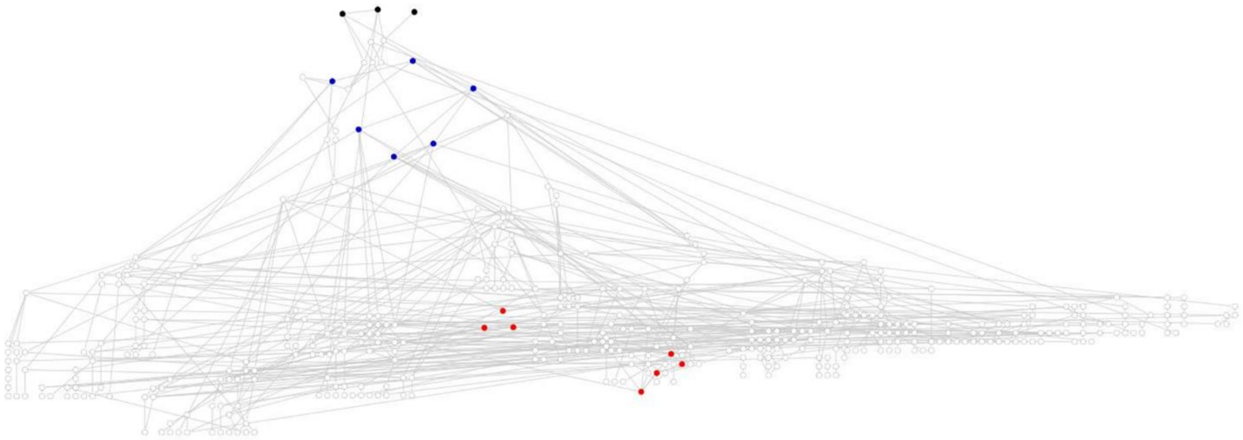

Figure S4. related to figure 5 | Flux map for the model of Cog4KO HEK293T cells.

(A) Total flux map for all enzymatic glycan processing reactions for 10,000 input glycans occurring during the simulation of the Cog4KO HEK293T glycan profile. Blue dots represent the substrates of the six reactions with the highest fluxes of all FUT8 catalysed reactions. Red dots represent the most abundant fucosylated glycans in the observed glycan profile of these cells. Black dots represent the three precursor ER glycans.

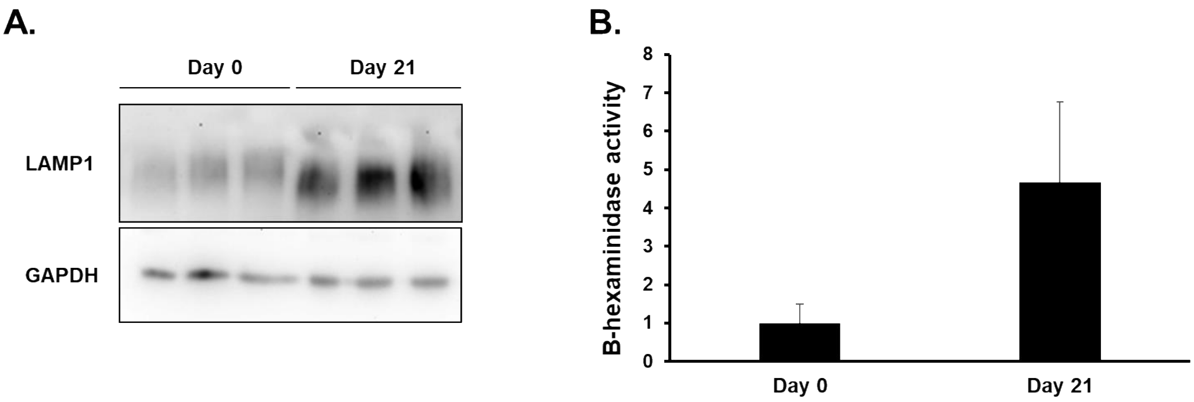

Figure S5. related to figure 6 | Lysosomal content increases upon osteogenesis.

(A) Western blot of LAMP1 in day 0 MSCs and Day 21 osteoblasts. (B) Beta-hexosaminidase activity of day 0 MSCs (n =3) and Day 21 osteoblasts (n = 2). Error bars are standard deviation.

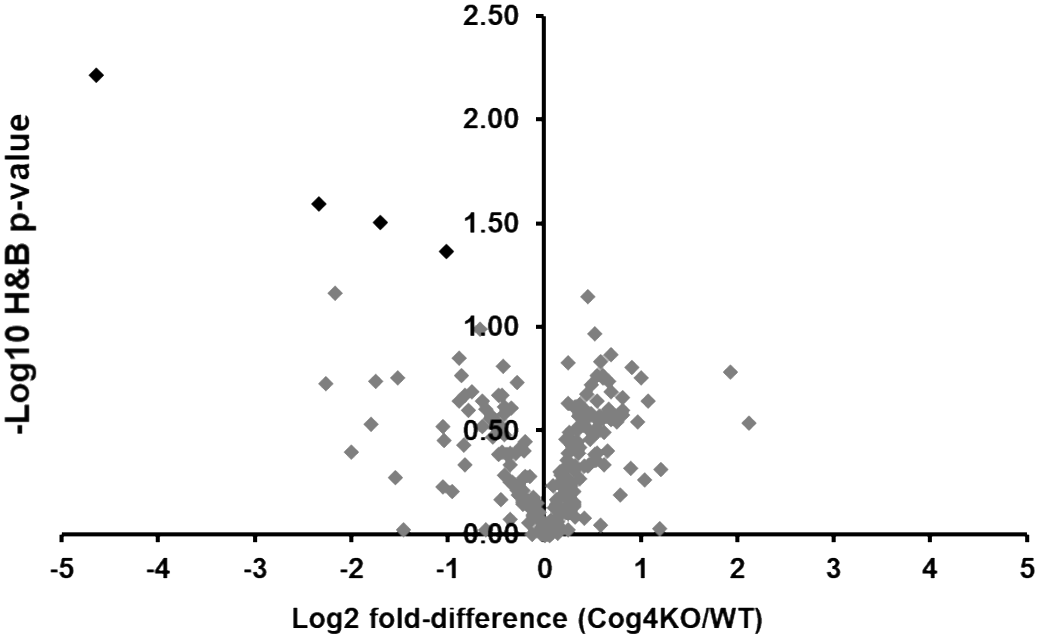

Figure S6. related to figure 4 | Abundance of membrane proteins in WT and Cog4KO HEK293T cells.

Volcano plot representing the ratio of abundance for transmembrane proteins (not including Golgi associated proteins) in WT vs Cog4KO HEK293T cells determined by mass spectrometry. The four black points represent the only proteins which show a significant difference in the Cog4KO HEK293T cells when compared with the WT. Please refer to supplementary table 3 for raw data.

Enzyme Name	Enzyme Target	Enzyme Result
**MANI (1)**	1Man2.1Man:	1Man:
**MANI (2)**	GlcNAc4.1GlcNAc4.1Man(3.1ManSS2.1Man:)_m6.1Man(3.1Man:)_m6.1Man:	GlcNAc4.1GlcNAc4.1Man(3.1Man:)_m6.1Man(3.1Man:)_m6.1Man:
**Endo-mannosidase**	GlcNAc4.1GlcNAc4.1Man(3.1ManSS2.1Man2.1Man3.1Gluc:)_m6.1Man(3.1Man2.1ManGG:)_m6.1Man2.1ManGG:	GlcNAc4.1GlcNAc4.1Man(3.1ManSS2.1Man:)_m6.1Man(3.1Man2.1Man:)_m6.1Man2.1Man:
**MAN2**	Man(3.1Man2.1GlcNAc:)_m6.1Man(3.1Man:)_m6.1Man:	Man(3.1Man2.1GlcNAc:)_m6.1Man:
**MGAT 1**	4.1Man(3.1Man:)_m6.1Man(3.1Man:)_m6.1Man:	4.1Man(3.1Man2.1GlcNAc:)_m6.1Man(3.1Man:)_m6.1Man:
**MGAT 2 (1)**	GlcNAc:)_m6.1Man:	GlcNAc:)_m6.1Man2.1GlcNAc:
**MGAT 2 (2)**	GlcNAc4.1Gal:)_m6.1Man:	GlcNAc4.1Gal:)_m6.1Man2.1GlcNAc:
**MGAT 2 (3)**	GlcNAc4.1Gal6.2Sia:)_m6.1Man:	GlcNAc4.1Gal6.2Sia:)_m6.1Man2.1GlcNAc:
**MGAT 4**	3.1Man2.1GlcNAc:	3.1Man(2.1GlcNAc:)_m4.1GlcNAc:
**MGAT 5**	6.1Man2.1GlcNAc:	6.1Man(2.1GlcNAc:)_m6.1GlcNAc:
**FUT8 (1)**	GlcNAc4.1GlcNAc4.1Man(3.1Man2.1GlcNAc:)_m6.1Man	GlcNAc(6.1Fuc:)_g4.1GlcNAc4.1Man(3.1Man2.1GlcNAc:)_m6.1Man
**FUT8 (2)**	GlcNAc4.1GlcNAc4.1Man(3.1Man(2.1GlcNAc:)_m4.1GlcNAc	GlcNAc(6.1Fuc)_g4.1GlcNAc4.1Man(3.1Man(2.1GlcNAc:)_m4.1GlcNAc
**FUT8 (3)**	GlcNAc4.1GlcNAc4.1Man(3.1Man2.1GlcNAc4.1Gal:)_m6.1Man	GlcNAc(6.1Fuc:)_g4.1GlcNAc4.1Man(3.1Man2.1GlcNAc4.1Gal:)_m6.1Man
**FUT8 (4)**	GlcNAc4.1GlcNAc4.1Man(3.1Man2.1GlcNAc4.1Gal6.2Sia:)_m6.1Man	GlcNAc(6.1Fuc:)_g4.1GlcNAc4.1Man(3.1Man2.1GlcNAc4.1Gal6.2Sia:)_m6.1Man
**FUT8 (5)**	GlcNAc4.1GlcNAc4.1Man(3.1Man(2.1GlcNAc4.1Gal:)_m4.1GlcNAc	GlcNAc(6.1Fuc:)_g4.1GlcNAc4.1Man(3.1Man(2.1GlcNAc4.1Gal:)_m4.1GlcNAc
**FUT8 (6)**	GlcNAc4.1GlcNAc4.1Man(3.1Man(2.1GlcNAc4.1Gal6.2Sia:)_m4.1GlcNAc	GlcNAc(6.1Fuc:)_g4.1GlcNAc4.1Man(3.1Man(2.1GlcNAc4.1Gal6.2Sia:)_m4.1GlcNAc
**FUT8 (7)**	GlcNAc4.1GlcNAc4.1Man(3.1Man:)_m6.1Man(3.1Man:)_m6.1Man:	GlcNAc(6.1Fuc:)_g4.1GlcNAc4.1Man(3.1Man:)_m6.1Man(3.1Man:)_m6.1Man:

**FUT8 off rate**	F:	:
**Antenna FUT**	GlcNAc4.1Gal	GlcNAc(3.1Fuc:)_g4.1Gal
**GalT (1)**	2.1GlcNAc:)_m6.1Man:	2.1GlcNAc4.1Gal:)_m6.1Man:
**GalT (2)**	2.1GlcNAc:)_m6.1Man(3.1Man:)_m6.1Man:	2.1GlcNAc4.1Gal:)_m6.1Man(3.1Man:)_m6.1Man:
**GalT (3)**	2.1GlcNAc:)_m6.1Man2.1GlcNAc	2.1GlcNAc4.1Gal:)_m6.1Man2.1GlcNAc
**GalT (4)**	6.1Man2.1GlcNAc:	6.1Man2.1GlcNAc4.1Gal:
**GalT (5)**	2.1GlcNAc:)_m6.1Man(2.1GlcNAc	2.1GlcNAc4.1Gal:)_m6.1Man(2.1GlcNAc
**GalT (6)**	2.1GlcNAc:)_m4.1GlcNAc	2.1GlcNAc4.1Gal:)_m4.1GlcNAc
**GalT (7)**	6.1Man(2.1GlcNAc:	6.1Man(2.1GlcNAc4.1Gal:
**GalT (8)**	4.1GlcNAc:	4.1GlcNAc4.1Gal:
**GalT (9)**	6.1GlcNAc:	6.1GlcNAc4.1Gal:
**SiaT (1)**	3.1Man2.1GlcNAc4.1Gal:	3.1Man2.1GlcNAc4.1Gal6.2Sia:
**SiaT (2)**	3.1Man(2.1GlcNAc4.1Gal:)_m4.1GlcNAc	3.1Man(2.1GlcNAc4.1Gal6.2Sia:)_m4.1GlcNAc
**SiaT (3)**	.1Gal:	.1Gal6.2Sia:
**OM quench**	1Man2.1Man:	1Man2.1ManP:

Table S1. related to figure 1 | Enzymes and enzyme rules used for wild type cell line modelling.

We worked with the assumption that mainly glycans containing one fucose were fucosylated on the core by FUT8. *N*-glycans that were doubly fucosylated were assumed to be fucosylated on the core and antenna. The glycan profiling data used in this work does not distinguish between the different isoenzymes that fucosylated on the antennae hence the annotation antenna (ant) fucosylation, which groups together all the fucosylation that we assume is on antennae rather than in the core.

Parameter	Initial value	Fitted HeLa	Fitted HEK293T	Fitted hTERT MSC
**$Man_6GlcNAc_2$ to $Man_5GlcNAc_2$**	10	6.27	8.87	11.40
**Fucosylation of $Man_5GlcNAc_2$**	20	1.24[a]	2.90[b]	-
**Sialylation of separate branches**	1	-	2.18	1.82
**Galactosylation of bi- vs tri- and tetra-antennary glycans**	1	-	2.81	1.99

Table S2. related to figure 2 | Enzymatic substrate specificities.

Scaling factors included in the modelling, to account for substrate-specific rate differences for selected enzymes. Numbers shown are dividing scale factors; for example, the reaction converting $Man_6GlcNAc_2$ to $Man_5GlcNAc_2$ is 6.27 times slower than other MAN1-catalysed reactions for HeLa cells.

[a,b] The fitted value for the fucosylation of $Man_5GlcNAc_2$ is for the swainsonine-treated condition. See also Figure S2.

Table S3. related to figure 4 | Crude total membrane proteomics.

Comparison of protein levels in WT and COG4KO HEK293T cells**.** Four proteins (highlighted) change significantly between the two cell types but otherwise the overall membrane-proteomes are similar**.** Golgi proteins were removed to account for the fact that the different Golgi morphology in the mutant could potentially cause selective loss of the organelle during digitonin treatment. This is an Excel file linked to the online version of this paper.

Enzyme Name	Effective enzymatic rate (cisterna 1)	Effective enzymatic rate (cisterna 2)	Effective enzymatic rate (cisterna 3)
**MAN1**	0.233496705	0.527139663	0.178111358
**MAN1(2)**	0.037217926	0.084022791	0.028389845
**EndoMAN**	0.029461455	0.003994726	0.004010006
**MAN2**	0.453718821	3.451994737	0.568873221
**MGAT1**	0.01393476	0.304813621	0.009163032
**MGAT2(1)**	1.798471895	12.77035395	1.700470263
**MGAT2(2)**	1.798471895	12.77035395	1.700470263
**MGAT2(3)**	1.798471895	12.77035395	1.700470263
**MGAT4**	0.004194392	0.031407158	0.004211912
**MGAT5**	0.020214963	0.307998276	0.020739824
**FUT8(1)**	0.049015259	0.412099132	0.053306307
**FUT8(2)**	0.049015259	0.412099132	0.053306307
**FUT8(3)**	0.049015259	0.412099132	0.053306307
**FUT8(4)**	0.049015259	0.412099132	0.053306307
**FUT8(5)**	0.049015259	0.412099132	0.053306307
**FUT8(6)**	0.049015259	0.412099132	0.053306307
**FUT8(7)**	0.000147627	0.001514319	0.000146666
**GalT(1)**	0.451799263	0.448824895	3.148618158
**GalT(2)**	0.451799263	0.448824895	3.148618158
**GalT(3)**	0.451799263	0.448824895	3.148618158
**GalT(4)**	0.451799263	0.448824895	3.148618158
**SiaT**	0.006008948	0.006231213	0.127969208
**OM quench**	0.125711779	0.014646036	0.014101402
	0.710849474[1]	0.009048474[2]	5.279178421[3]

Table S4. related to figure 2 | WT HeLa fitted parameters.

Table representing the fitted parameter values for WT HeLa cells. [1]proportion of $Man_8GlcNAc_2$ as input glycan, [2]proportion of $GluMan_9GlcNAc_2$ as input glycan, [3]transit time per cisterna.

Enzyme Name	Effective enzymatic rate (cisterna 1)	Effective enzymatic rate (cisterna 2)	Effective enzymatic rate (cisterna 3)	Effective enzymatic rate (cisterna 4)
**MAN1**	0.194485087	1.7516927	0.22349944	0.022924432
**MAN1(2)**	0.021928699	0.197507902	0.025200142	0.002584789
**EndoMAN**	0.030639145	0.005333927	0.004076729	0.000423947
**MAN2**	0.50131336	2.9467012	1.914476833	0.547513267
**MGAT1**	0.007119538	0.636804333	0.098816213	0.009268861
**MGAT2(1)**	1.796796067	6.5077812	5.896882133	1.7318214
**MGAT2(2)**	1.796796067	6.5077812	5.896882133	1.7318214
**MGAT2(3)**	1.796796067	6.5077812	5.896882133	1.7318214
**MGAT4**	0.004176106	0.02006288	0.019514897	0.004981294
**MGAT5**	0.020047691	0.168627987	0.24528994	0.031690672
**FUT8(1)**	0.049176632	0.177289367	0.071655067	0.04276546
**FUT8(2)**	0.049176632	0.177289367	0.071655067	0.04276546
**FUT8(3)**	0.049176632	0.177289367	0.071655067	0.04276546
**FUT8(4)**	0.049176632	0.177289367	0.071655067	0.04276546
**FUT8(5)**	0.049176632	0.177289367	0.071655067	0.04276546
**FUT8(6)**	0.049176632	0.177289367	0.071655067	0.04276546
**FUT8(7)**	0.002713389	0.009782188	0.003953668	0.002359644
**FUT8OFF**	0.099446587	0.099446587	0.099446587	0.099446587
**Ant FUT**	0.000999622	0.014081887	0.011049582	0.001026963
**GalT(1)**	0.010020635	0.072018267	0.2061617	0.25531468
**GalT(2)**	0.010020635	0.072018267	0.2061617	0.25531468
**GalT(3)**	0.010020635	0.072018267	0.2061617	0.25531468
**GalT(4)**	0.010020635	0.072018267	0.2061617	0.25531468
**GalT(5)**	0.003562637	0.025604659	0.073296684	0.090772046
**GalT(6)**	0.003562637	0.025604659	0.073296684	0.090772046
**GalT(7)**	0.003562637	0.025604659	0.073296684	0.090772046
**GalT(8)**	0.003562637	0.025604659	0.073296684	0.090772046
**GalT(9)**	0.003562637	0.025604659	0.073296684	0.090772046
**SiaT(1)**	0.006000835	0.006713236	0.084122005	0.09841716
**SiaT(2)**	0.006000835	0.006713236	0.084122005	0.09841716
**SiaT(3)**	0.002754859	0.003081907	0.038618664	0.045181272
**OM quench**	0.052862328	0.011772589	0.014043215	0.001410821
	0.1132855[1]	0.030996753[2]	7.2580224[3]	

Table S5. related to figure 2 | WT HEK293T fitted parameters.

Table representing the fitted parameter values for WT HEK293T cells. [1]proportion of $Man_8GlcNAc_2$ as input glycan, [2]proportion of $GlcMan_9GlcNAc_2$ as input glycan, [3]transit time per cisterna.

Enzyme Name	Effective enzymatic rate (cisterna 1)	Effective enzymatic rate (cisterna 2)	Effective enzymatic rate (cisterna 3)	Effective enzymatic rate (cisterna 4)
**MAN1**	0.15	1.757304643	0.154082229	0.024309597
**MAN1(2)**	0.013169447	0.154202283	0.01352061	0.002133151
**EndoMAN**	0.030250348	0.005568547	0.003948526	0.00041535
**MAN2**	0.506190971	4.903260857	2.659008286	0.545422786
**MGAT1**	0.00693554	0.5	0.1	0.009975619
**MGAT2(1)**	0.179587836	0.564087143	0.727762607	0.248050429
**MGAT2(2)**	0.179587836	0.564087143	0.727762607	0.248050429
**MGAT2(3)**	0.179587836	0.564087143	0.727762607	0.248050429
**MGAT4**	0.004190141	0.014352356	0.018409193	0.005405872
**MGAT5**	0.020134409	0.057334779	0.076209429	0.030761723
**FUT8(1)**	0.048188871	0.15043975	0.057443964	0.057784225
**FUT8(2)**	0.048188871	0.15043975	0.057443964	0.057784225
**FUT8(3)**	0.048188871	0.15043975	0.057443964	0.057784225
**FUT8(4)**	0.048188871	0.15043975	0.057443964	0.057784225
**FUT8(5)**	0.048188871	0.15043975	0.057443964	0.057784225
**FUT8(6)**	0.048188871	0.15043975	0.057443964	0.057784225
**FUT8(7)**	0.002654511	0.008287059	0.003164333	0.003183077
**FUT8OFF**	0.082556143	0.082556143	0.082556143	0.082556143
**Ant FUT**	0.000998262	0.013721026	0.0007	0.0007
**GalT(1)**	0.010078676	0.065822614	0.784380357	0.483927857
**GalT(2)**	0.010078676	0.065822614	0.784380357	0.483927857
**GalT(3)**	0.010078676	0.065822614	0.784380357	0.483927857
**GalT(4)**	0.010078676	0.065822614	0.784380357	0.483927857
**GalT(5)**	0.005056857	0.033025725	0.393553645	0.242805127
**GalT(6)**	0.005056857	0.033025725	0.393553645	0.242805127
**GalT(7)**	0.005056857	0.033025725	0.393553645	0.242805127
**GalT(8)**	0.005056857	0.033025725	0.393553645	0.242805127
**GalT(9)**	0.005056857	0.033025725	0.393553645	0.242805127
**SiaT(1)**	0.006077692	0.006680604	0.04679148	0.034442945
**SiaT(2)**	0.006077692	0.006680604	0.04679148	0.034442945
**SiaT(3)**	0.003315519	0.003644421	0.025525814	0.018789408
**OM quench**	0.03	0.01252273	0.013914026	0.001323202
	0.3[1]	0.017735158[2]	10.03370857[3]	

Table S6. related to figure 6 | Y101 hTERT MSC fitted parameters.

Table representing the fitted parameter values for hTERT-MSCs (Y101s). [1]proportion of $Man_8GlcNAc_2$ as input glycan, [2]proportion of $GlcMan_9GlcNAc_2$ as input glycan, [3]transit time per cisterna.

Enzyme Name	Effective enzymatic rate (cisterna 1)	Effective enzymatic rate (cisterna 2)	Effective enzymatic rate (cisterna 3)	Effective enzymatic rate (cisterna 4)
**MAN1**	0.307558695	1.7744673	0.17476228	0.026185174
**MAN1(2)**	0.030061662	0.173441485	0.017081763	0.002559413
**EndoMAN**	0.033662145	0.00588473	0.004029459	0.000402867
**MAN2**	0.5079001	6.093339	3.377208	0.587262
**MGAT1**	0.007114036	0.6225316	0.079531065	0.009841085
**MGAT2(1)**	0.18019222	0.46439071	0.7227887	0.26702385
**MGAT2(2)**	0.18019222	0.46439071	0.7227887	0.26702385
**MGAT2(3)**	0.18019222	0.46439071	0.7227887	0.26702385
**MGAT4**	0.004192536	0.009751601	0.017818555	0.005511443
**MGAT5**	0.019955005	0.029678331	0.04895692	0.032459256
**FUT8(1)**	0.047849834	0.154176175	0.0523304	0.05802216
**FUT8(2)**	0.047849834	0.154176175	0.0523304	0.05802216
**FUT8(3)**	0.047849834	0.154176175	0.0523304	0.05802216
**FUT8(4)**	0.047849834	0.154176175	0.0523304	0.05802216
**FUT8(5)**	0.047849834	0.154176175	0.0523304	0.05802216
**FUT8(6)**	0.047849834	0.154176175	0.0523304	0.05802216
**FUT8(7)**	0.002452617	0.007902537	0.002682275	0.002974015
**FUT8OFF**	0.092797335	0.092797335	0.092797335	0.092797335
**Ant FUT**	0.000995563	0.01234311	0.000676167	0.000579693
**GalT(1)**	0.010083057	0.05723082	1.14757235	0.53553684
**GalT(2)**	0.010083057	0.05723082	1.14757235	0.53553684
**GalT(3)**	0.010083057	0.05723082	1.14757235	0.53553684
**GalT(4)**	0.010083057	0.05723082	1.14757235	0.53553684
**GalT(5)**	0.005173889	0.029366679	0.588850366	0.274798416
**GalT(6)**	0.005173889	0.029366679	0.588850366	0.274798416
**GalT(7)**	0.005173889	0.029366679	0.588850366	0.274798416
**GalT(8)**	0.005173889	0.029366679	0.588850366	0.274798416
**GalT(9)**	0.005173889	0.029366679	0.588850366	0.274798416
**SiaT(1)**	0.006091488	0.007073644	0.041135007	0.03149241
**SiaT(2)**	0.006091488	0.007073644	0.041135007	0.03149241
**SiaT(3)**	0.002920068	0.003390883	0.019718831	0.015096473
**OM quench**	0.013152243	0.012452489	0.014328323	0.001424251
	0.301190675[1]	0.012826073[2]	12.091441[3]	

Table S7. related to figure 6 | Osteoblast fitted parameters.

Table representing the fitted parameter values for osteoblasts derived from hTERT-MSCs (Y101s). [1]proportion of $Man_8GlcNAc_2$ as input glycan, [2]proportion of $GlcMan_9GlcNAc_2$ as input glycan, [3]transit time per cisterna.
