# Supplementary material for: Modeling Glycan Processing Reveals Golgi-Enzyme Homeostasis upon Trafficking Defects and Cellular Differentiation
Source: Cell Rep. 2019 Apr 23;27(4):1231–1243.e6. doi: 10.1016/j.celrep.2019.03.107 (PMC6486481; doi:10.1016/j.celrep.2019.03.107)
Supplement: Document S2. Article plus Supplemental Information [file mmc3.pdf]

Cell Reports

Article

Modeling Glycan Processing Reveals Golgi-Enzyme Homeostasis upon Trafficking Defects and Cellular Differentiation

Graphical Abstract

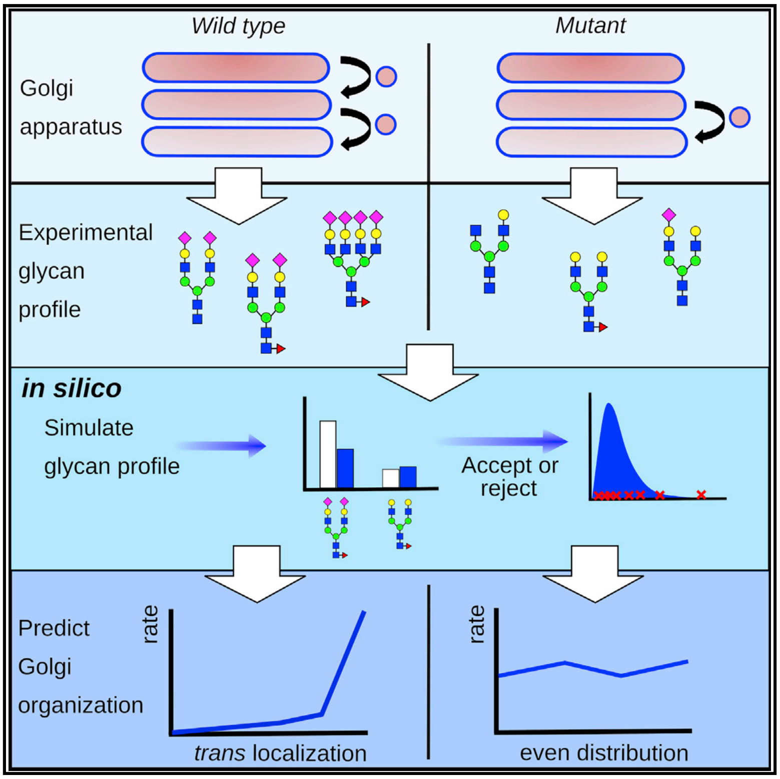

Highlights

- Developed a stochastic model of *N*-glycosylation coupled with Bayesian fitting
- Validated predicted changes of Golgi organization in trafficking mutants
- Model pinpointed functionally relevant glycan alterations in osteogenesis

Authors

Peter Fisher, Hannah Spencer, Jane Thomas-Oates, A. Jamie Wood, Daniel Ungar

Correspondence

jane.thomas-oates@york.ac.uk (J.T.-O.),
jamie.wood@york.ac.uk (A.J.W.),
dani.ungar@york.ac.uk (D.U.)

In Brief

A biologically relevant model of glycan processing was developed for predicting changes in enzyme organization within the Golgi. Fisher et al. predict and experimentally validate changes in Golgi-enzyme levels and localizations due to perturbation to intra-Golgi trafficking, and they describe a functionally important change in glycan-branching activity during osteogenesis.

Fisher et al., 2019, Cell Reports *27*, 1231–1243
April 23, 2019 © 2019 The Authors.
https://doi.org/10.1016/j.celrep.2019.03.107

CellPress

OPEN ACCESS
CellPress

Modeling Glycan Processing Reveals Golgi-Enzyme Homeostasis upon Trafficking Defects and Cellular Differentiation

Peter Fisher,[1,2] Hannah Spencer,[1,2] Jane Thomas-Oates,[2,*] A. Jamie Wood,[1,3,*] and Daniel Ungar[1,4,*]
[1]Department of Biology, University of York, York YO10 5DD, UK
[2]Department of Chemistry and Centre of Excellence in Mass Spectrometry, University of York, York YO10 5DD, UK
[3]Department of Mathematics, University of York, York YO10 5DD, UK
[4]Lead Contact
*Correspondence: jane.thomas-oates@york.ac.uk (J.T.-O.), jamie.wood@york.ac.uk (A.J.W.), dani.ungar@york.ac.uk (D.U.)
https://doi.org/10.1016/j.celrep.2019.03.107

SUMMARY

The decoration of proteins by carbohydrates is essential for eukaryotic life yet heterogeneous due to a lack of biosynthetic templates. This complex carbohydrate mixture—the glycan profile—is generated in the compartmentalized Golgi, in which level and localization of glycosylation enzymes are key determinants. Here, we develop and validate a computational model for glycan biosynthesis to probe how the biosynthetic machinery creates different glycan profiles. We combined stochastic modeling with Bayesian fitting that enables rigorous comparison to experimental data despite starting with uncertain initial parameters. This is an important development in the field of glycan modeling, which revealed biological insights about the glycosylation machinery in altered cellular states. We experimentally validated changes in *N*-linked glycan-modifying enzymes in cells with perturbed intra-Golgi-enzyme sorting and the predicted glycan-branching activity during osteogenesis. Our model can provide detailed information on altered biosynthetic paths, with potential for advancing treatments for glycosylation-related diseases and glyco-engineering of cells.

INTRODUCTION

Glycosylation is a ubiquitous post-translational modification in eukaryotes. It plays roles ranging from protein stability (Waetzig et al., 2010) through cell adhesion (Zhao et al., 2008) to complex physiological traits like antibody-dependent cellular cytotoxicity (Ferrara et al., 2006; Shields et al., 2002). *N*-linked glycosylation is initiated in the endoplasmic reticulum (ER), with subsequent glycan processing during transport through the Golgi. Within the Golgi, an interplay of glycosidase-mediated mannose trimming and monosaccharide additions via glycosyltransferases (Figure 1A) generates *N*-glycans classed as oligomannose (five to nine mannoses), hybrid (at least one *N*-acetylglucosamine [GlcNAc]-initiated antenna and five mannoses), and complex (up to five GlcNAc-seeded antennae). The GlcNAc-seeded antennae in complex glycans can be extended through galactosylation and capped by the often-functional sialic acid (Christie et al., 2008; Scott and Panin, 2014). Another functionally important modification is fucosylation, which can occur on the chain-initiating GlcNAc (core fucosylation) or the antennae (Hemmerich et al., 1994). In the absence of a template, and due to the limited time spent in the Golgi, competition among enzymatic modifications generates a heterogeneous glycan mixture (Stanley and Sudo, 1981).

Glycosylation enzymes are non-uniformly distributed between Golgi cisternae (Dunphy and Rothman, 1983; Rabouille et al., 1995). The levels and distributions of enzymes are cell type specific, and together they determine a given cell's glycan profile (Fisher and Ungar, 2016). Enzyme distributions are maintained through sorting via COPI-coated retrograde vesicles (Harris and Waters, 1996) that must be correctly targeted to the relevant cisterna or cisternae. The conserved oligomeric Golgi (COG) complex is involved in COPI-vesicle targeting at the Golgi apparatus through its ability to self-assemble (Willett et al., 2016) and interact with other trafficking proteins (Fukuda et al., 2008; Miller et al., 2013; Willett et al., 2013). COG defects have been shown to result in aberrant glycosylation in model systems (Abdul Rahman et al., 2014; Bailey Blackburn et al., 2016; Belloni et al., 2012; Pokrovskaya et al., 2011; Struwe and Reinhold, 2012; Whyte and Munro, 2001) and in an expanding group of genetic disorders known as congenital disorders of glycosylation (CDGs) (Kodera et al., 2015; Zeevaert et al., 2008).

Mesenchymal stromal cells (MSCs) are a valuable model for studying glycan function. They can differentiate into several cell types, and the Y101 immortalized human MSC line readily differentiates into osteoblasts under suitable growth conditions (James et al., 2015). The glycan profile of Y101-derived osteoblasts is markedly different from that of the parent MSC lines (Wilson et al., 2016). Glycosylation has been shown to modulate the ability of this MSC line to undergo osteogenesis (Wilson et al., 2018). Specifically, inhibition of mannosidase I (MAN1), which limits *N*-glycans to their oligomannose form, increases differentiation. Yet it is unclear which glycan structures are modulating differentiation (Wilson et al., 2018).

Established kinetic models of glycosylation, based on ordinary differential equations (ODEs) (Krambeck and Betenbaugh, 2005;

This is an open access article under the CC BY license (http://creativecommons.org/licenses/by/4.0/).

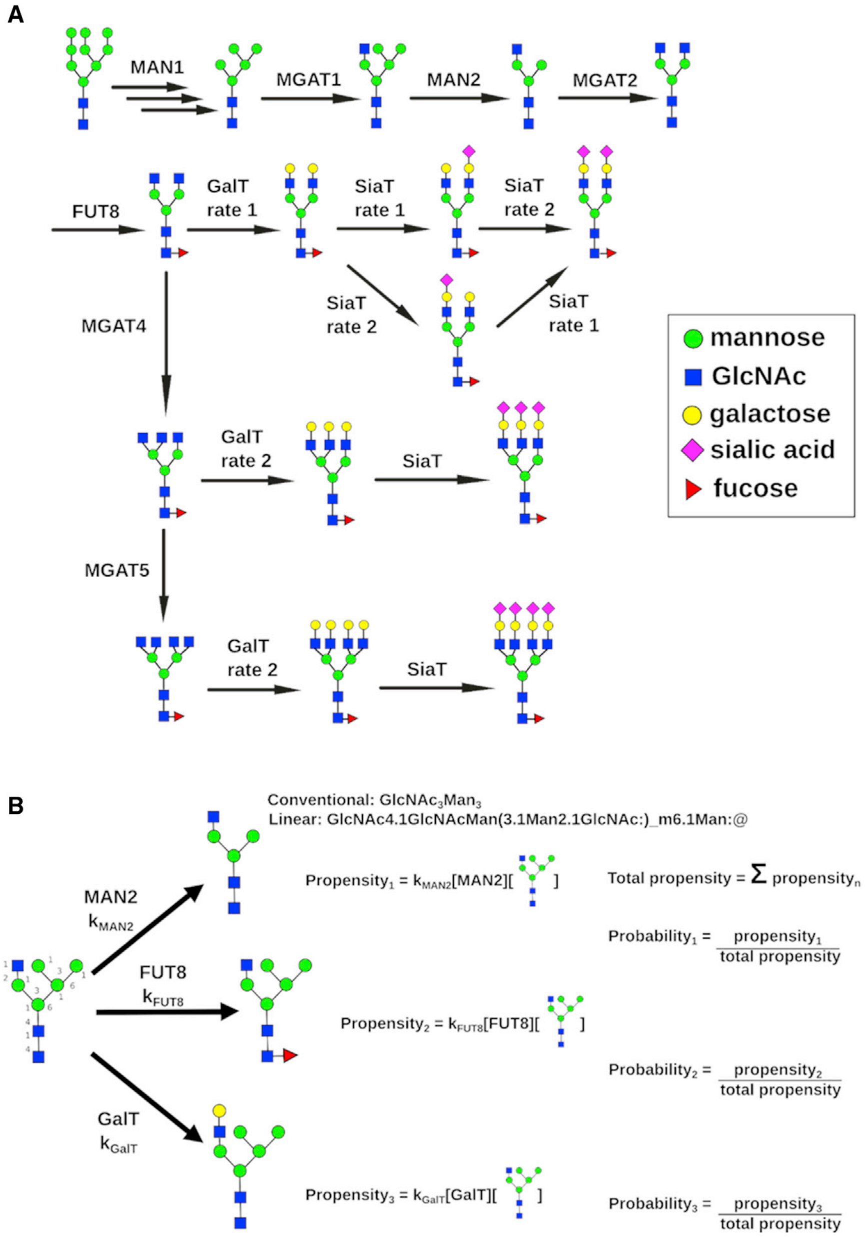

Figure 1. Modeling *N*-Linked Glycosylation in Mammalian Cells

(A) Example of the sequential nature of the *N*-linked glycosylation pathway in mammalian cells. MAN1 trims oligomannose glycans before conversion to the hybrid class through the action of MGAT1. Before galactose is added to the complex branch of the hybrid structure, the removal of two mannose residues by MAN2 can convert hybrid into complex glycans. Fucosylation on the antennae of complex glycans (ant Fut) can be catalyzed by several fucosyltransferases that are grouped for the simulation of glycosylation reactions. The sialylation rate for the different branches of bi-antennary glycans was modeled separately to account for potentially different rates.

(B) Schematic representation of the stochastic simulation algorithm (SSA) used to simulate glycosylation reactions. The propensity for each competing reaction is used to calculate the probability of each reaction occurring. Time is then advanced by δt, which is drawn from an exponential distribution with a mean equal to $1/\textit{total propensity}$. An example of the linear notation used to define a glycan structure, which was used for substrate recognition and modification following a reaction by the modeling software, is also included. Each monosaccharide residue is defined, along with the link as a linear string. Brackets represent separate branches, and colons represent the terminus of each branch. These strings can be recognized by enzyme rules in the SSA. The conventional notations for the glycan structures are shown for comparison.

See also Figure S1 and Table S1.

Krambeck et al., 2009; Umaña and Bailey, 1997), assume that the essential dynamics of glycan biosynthesis are appropriately captured by this deterministic approach. Other models based on ODEs have explored the role of multiple compartments in glycan biosynthesis (Hossler et al., 2007; Liu et al., 2008). For example, Hossler et al. (2007) modeled glycosylation in the context of vesicular transport and cisternal maturation of the Golgi apparatus and concluded that cisternal maturation was more likely to be the true mechanism, although it has been suggested that better approximations regarding modeling of the cisternal maturation process could have been made (Krambeck et al., 2017). These models of glycosylation are based on ODEs; however, the significant heterogeneity in glycan structures suggests that stochastic models (e.g., Spahn et al., 2016), explicitly incorporating molecular noise, may offer greater insight. This has been recognized in other aspects of biology (Eldar and Elowitz, 2010). Here we report development of a stochastic model of *N*-linked glycan processing and use Bayesian fitting (Csilléry et al., 2010) to measured glycan profiles to predict changes in Golgi-enzyme organization in three human model cell lines. In our model, we started with the assumption that the enzymatic rates are not substrate specific, only enzyme specific, in contrast to ODE models. Substrate specificity for a limited set of reactions was introduced when required as typical of deterministic models. Our model makes experimentally testable predictions about the altered levels and localizations of enzymes in COG mutants and provides a testable hypothesis for the functional role of specific glycans during cell differentiation.

RESULTS

Model Development

A cell's highly heterogeneous *N*-glycan profile depends on the levels, enzymatic rates, and distributions of individual enzymes in the Golgi cisternae. We simulate the glycosylation reactions

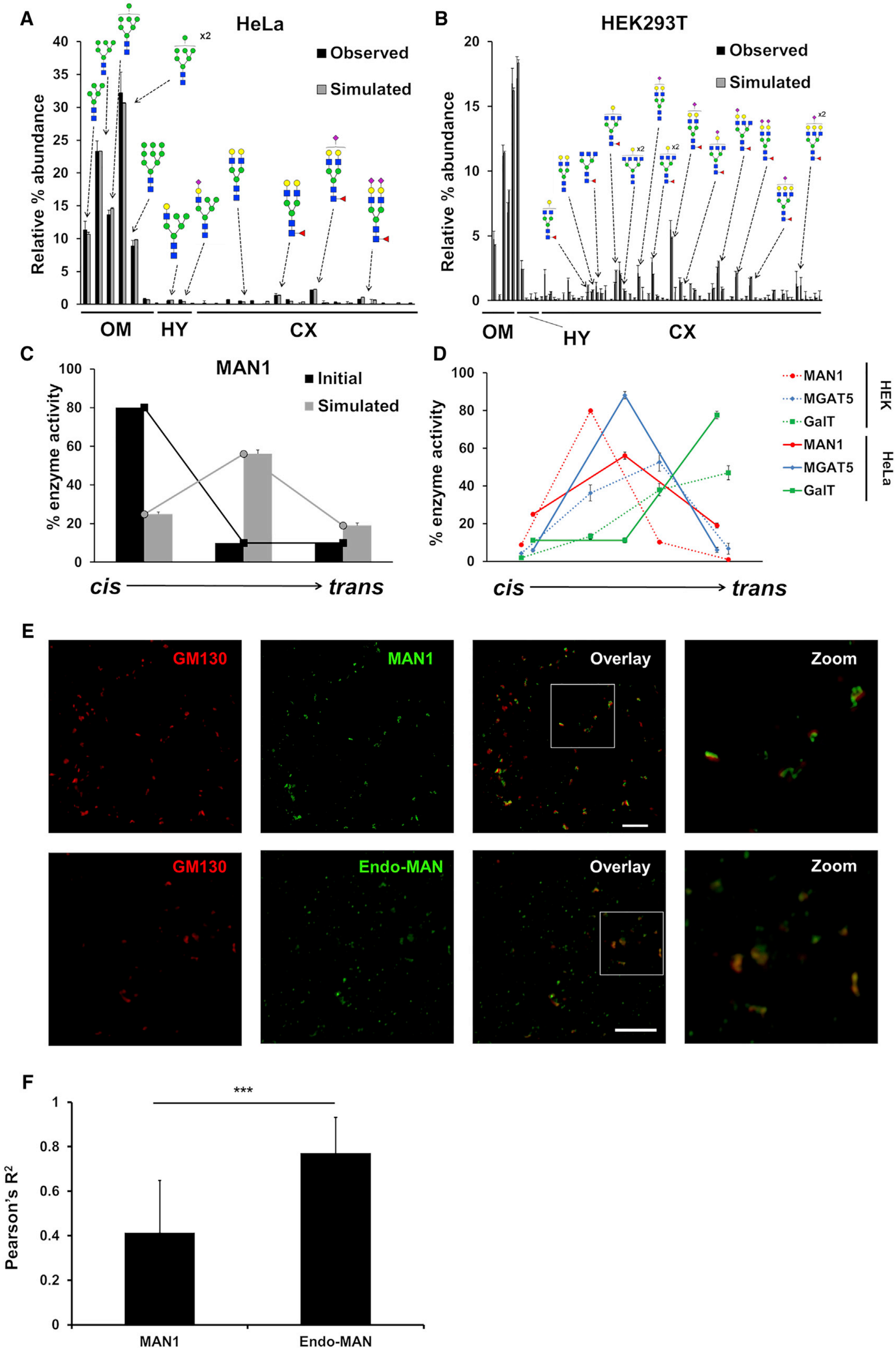

(legend on next page)

OPEN
ACCESS
CellPress

using a stochastic simulation algorithm (SSA) (Figure 1B) (Gibson and Bruck, 2000; Gillespie, 1976), in contrast to the differential equation approach used previously (Krambeck and Betenbaugh, 2005; Krambeck et al., 2009). The SSA captures all possible reactions as discrete events, determined by a set of rules for each enzyme (Table S1) that dictate which substrates it is converting to which products. Each glycan is acted upon by a changing set of enzymes constrained by their available target sites; as new monosaccharides are added, acceptable target sites also change (Figure 1B). These reactions happen at random times and in a random order depending on the enzymatic rate and amount of each enzyme but independent of how the glycan substrate was generated.

The enzymatic reactions are implemented using string substitutions to add or remove monosaccharides on the glycans represented in a form of linear notation to build new structures (Figure 1B). The linear notation is similar to those previously used (Krambeck and Betenbaugh, 2005) and has been developed for computational convenience. All simulated glycan profiles are compared with experimental glycan profiles obtained mass spectrometrically from permethylated glycans. The use of MALDI mass spectrometry (MALDI-MS) of permethylated glycans has been demonstrated and accepted to provide relative quantification of glycans (Mehta et al., 2016; Orlando, 2010; Wada et al., 2007). To generate a computational glycan profile to be compared to experimental mass spectra, we simulate a large number (n = 10,000) of input glycans entering the Golgi one at a time. The glycans entering the simulated Golgi are drawn from a distribution of structures known to exit the ER (Man_8, Man_9, and Man_9Glc). As the glycan is processed by the SSA, it undergoes successive modifications until a time limit is reached and it passes to a new cisterna.

To determine a set of appropriate rates for the SSA based on the relative effective enzymatic rates of the glycan-modifying enzymes and their unique localizations within the Golgi stack, our computational method is parameterized using a Bayesian framework (Figure S1) (Marjoram et al., 2003). Throughout this work, we have adopted the term "effective enzymatic rate" to describe the composite nature of the parameter used in the modeling. We define the effective enzymatic rate as the product of the enzyme's protein level, the availability of its nucleotide-monosaccharide substrate, and its inherent chemical enzymatic rate. The effective enzymatic rate parameters are not direct measurements of enzyme rate constants but rather a composite of enzyme features. Only changes in these parameters are considered, not their absolute values; therefore, only relative, rather than absolute, glycan quantification is required for the modeling. The unique advantage of using a Bayesian framework is that it allows us to incorporate existing knowledge, no matter how tentative or precise, to construct a series of prior distributions for our parameters. This results in a broad distribution for largely unknown parameters or a sharp distribution centered around a single value for well-characterized parameters. This allows us to not require exact, well-defined enzymatic rates, protein levels, and localizations.

We challenge this modeling framework by comparing its output to experimental glycan profiles. This results in a fitting process whereby our initial prior distributions are systematically updated to give a new set of parameter distributions called posteriors. The shift between the priors and the posteriors both reflects clarifications of our existing knowledge during initial fitting and can capture real biological changes during empirical perturbation between cellular states.

Our experimental glycan profiles were collected from whole-cell lysates that contain a significant proportion of oligomannose glycans. Significant sources of these may be endo-lysosomal glycoproteins or recycled ER-resident proteins. Endo-lysosomal glycoproteins are predominantly decorated with oligomannose glycans due to the Man-6-phosphate (Man6P) targeting tag that prevents conversion to hybrid and complex glycans (Bieberich, 2014). ER-resident glycoproteins that are recycled from the Golgi may also contain oligomannose glycans that do not undergo further processing. Therefore, we included a parameter (oligomannose [OM] quench) that prevents oligomannose glycans from being further processed. The OM quench parameter is effectively a combination of the GlcNAc-1-phosphotransferase enzyme that initiates Man6P biosynthesis and the removal of ER glycoproteins from the Golgi via retrograde traffic to the ER. Modeling started with rules covering the set of reactions able to generate all possible glycans in a profile. To achieve high-quality fits to experimental glycan profiles, rules (detailed later) had to be refined and modified, justified by the existing literature. These refinements represent internal properties of the enzymes (e.g., substrate-specific rates) and were therefore modeled as scale factors that alter the effective enzymatic rates (Figure S2; Table S2).

Modeling *N*-Glycosylation in Mammalian Cell Lines

We first tested the model on the simple HeLa cell glycan profile (>90% oligomannose) and the more complex HEK293T glycan profile. The HeLa glycan profile could be reproduced *in silico* with a model distributing enzymes into three cisternae (Figure 2A; Table S4), the minimum number of cisternae required. Minimizing the cisterna number prevents excessive use of computational

Figure 2. Model Development for WT Mammalian Cell Lines

(A and B) Observed and simulated glycan profiles of whole-cell WT HeLa cells (A) and HEK293T cells (B). The glycan profile is simulated three times using the SSA, with the mean parameter values from all individual fitting runs used to generate an average glycan profile with error bars. For glycan profiles, the error bars are SEM for n = 3.

(C) Prior parameter distribution values for the MAN1 enzyme contrasted with posterior values following optimization of the MAN1 effective enzymatic rates. Initially, MAN1 was modeled as a *cis*-Golgi enzyme.

(D) Predicted distributions of selected enzymes following fitting. Error bars are SD for n = 20 (HeLa) and n = 15 (HEK293T) individual fitting procedures.

(E) Airyscan confocal micrographs of GM130 and MAN1 or endo-mannosidase in nocodazole-treated WT HEK293T cells. Scale bar is 5 μm.

(F) Pearson's correlation coefficients for GM130-MAN1 and GM130-endo-mannosidase. Pearson's correlation coefficients were calculated for each Golgi stack, and error bars are SD for n = 55 (MAN1) and n = 62 (endo-mannosidase) stacks. ***$p < 0.001$ for a Student's t test.

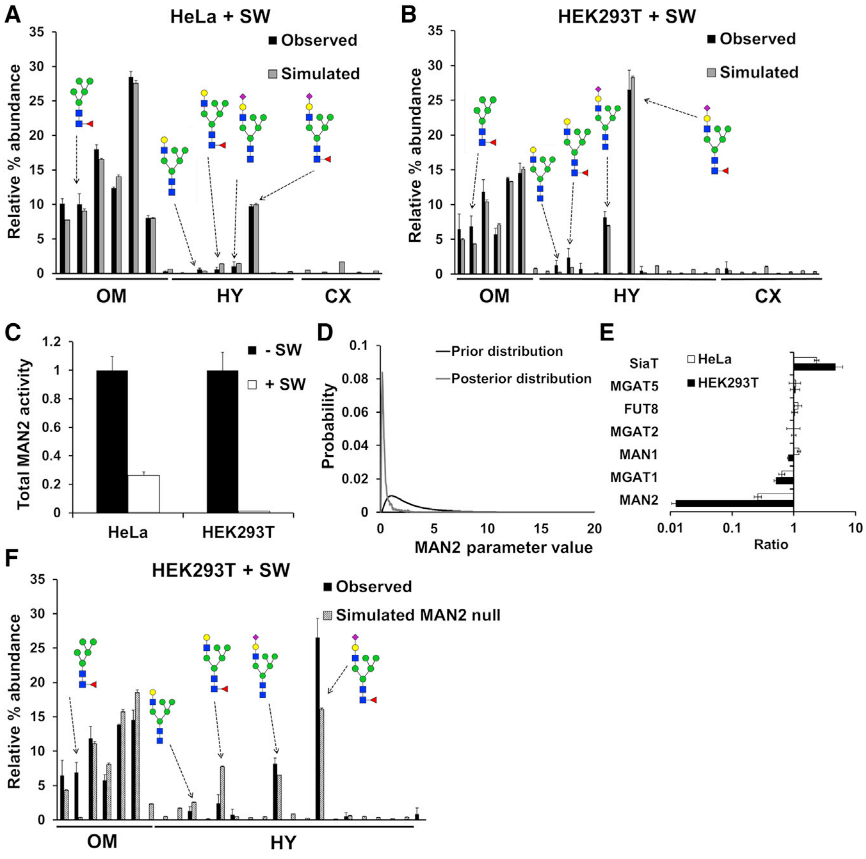

Figure 3. Validation of Fitting Methodology with Drug Treatment of Mammalian Cells

(A and B) Observed and simulated glycan profiles of HeLa cells (A) and HEK293T cells (B) following treatment with the MAN2 inhibitor swainsonine (SW). Error bars for glycan profiles are SEM for n = 3.

(C) Predicted total effective enzymatic rate of the MAN2 enzyme in both cell lines with and without swainsonine treatment normalized to the total MAN2 effective enzymatic rate modeled in the untreated cell lines.

(D) Prior and posterior distributions of the MAN2 effective enzymatic rate in the second cisterna of HEK293T cells.

(E) Total effective enzymatic rate changes upon swainsonine treatment for selected enzymes. Error bars are SD for n = 13 (HeLa) and n = 16 (HEK293T) individual fitting procedures.

(F) Observed swainsonine-treated HEK293T glycan profile and simulated glycan profile using the parameters fitted for untreated cells but with the MAN2 effective enzymatic rate set to 0.

See also Tables S4 and S5.

time. To fit the oligomannose glycan distribution, a scale factor was necessary to modify the rate for converting $Man_6GlcNAc_2$ to $Man_5GlcNAc_2$ as published (Bause et al., 1992; Lal et al., 1998); this was then used throughout the study (Figure S2; Table S2).

Our initial assumption based on the literature was that MAN1 is predominately *cis*-Golgi localized (Dunphy and Rothman, 1983; Marra et al., 2001). However, after fitting, the model predicted it spread over the whole stack, with a weak medial-Golgi preference (Figure 2C). Although often considered a *cis*-Golgi marker, MAN1 localization has been shown to vary across cell types (Velasco et al., 1993), consistent with our *in silico* finding. Furthermore, confocal microscopy revealed that MAN1 localizes adjacent to the *cis*-Golgi marker GM130 in HEK293T cells, in contrast to Golgi-endo-mannosidase that colocalizes with this marker (Figures 2E and 2F). This is consistent with our model's prediction that MAN1 is positioned away from the *cis* side and thus closer to the medial Golgi than the endo-mannosidase in the modeled cell lines (Table S4).

Fitting the HEK293T glycan profiles started from the fitted HeLa parameters, allowing comparison of the two cell lines. However, for a good HEK293T profile fit, a fourth model cisterna was required (Figure 2B; Table S5), likely due to this cell line's more complex glycan profile. Moreover, to achieve this fit, separate rates for the sialylation of galactoses on the 3.1Man and 6.1Man antennae (Barb et al., 2009; Joziasse et al., 1987), and galactosylation of bi- versus tri- and tetra-antennary glycans (Ramasamy et al., 2005), had to be introduced. These additions were presumably not required for fitting the HeLa cell data, because they mainly affect hybrid- and complex-type glycans, which are in low abundance in HeLa cells. In HEK293T cells, MAN1 is predicted to have a predominantly early-medial localization (Figure 2D; Table S5), in contrast to its medial location in HeLa cells, which is likely a consequence of the additional cisterna introduced to process more complex glycans.

To demonstrate that our model can make rational predictions, we treated both HeLa and HEK293T cells with the mannosidase II (MAN2) inhibitor swainsonine (Elbein et al., 1981). This results in strongly increased hybrid *N*-glycan content and generation of fucosylated $Man_5GlcNAc_2$. To allow for this fucosylation reaction, albeit at a lower rate than with other substrates (Crispin et al., 2006; Lin et al., 1994; Yang and Wang, 2016; Yang et al., 2017), a scale factor was added for fucosyltransferase 8 (FUT8) modifying $Man_5GlcNAc_2$ (Figure S2; Table S2). Starting with the parameter values obtained from fitting the glycan profiles of untreated cells, we fitted to the glycan profiles of swainsonine-treated HEK293T and HeLa cells by optimizing parameter values within the established modeling framework (Figures 3A and 3B). The model obtained predicts the expected large decrease in the effective enzymatic rate of MAN2 (Figures 3C and 3D).

The model also predicts an unexpected increase in effective sialylation rate in both cell lines (Figure 3E). The requirement for increased sialylation was confirmed by simulating a glycan profile using the original HEK293T parameters but with the MAN2 enzyme removed to account for the known effect of swainsonine. The resulting simulated profile only partially

OPEN ACCESS
CellPress

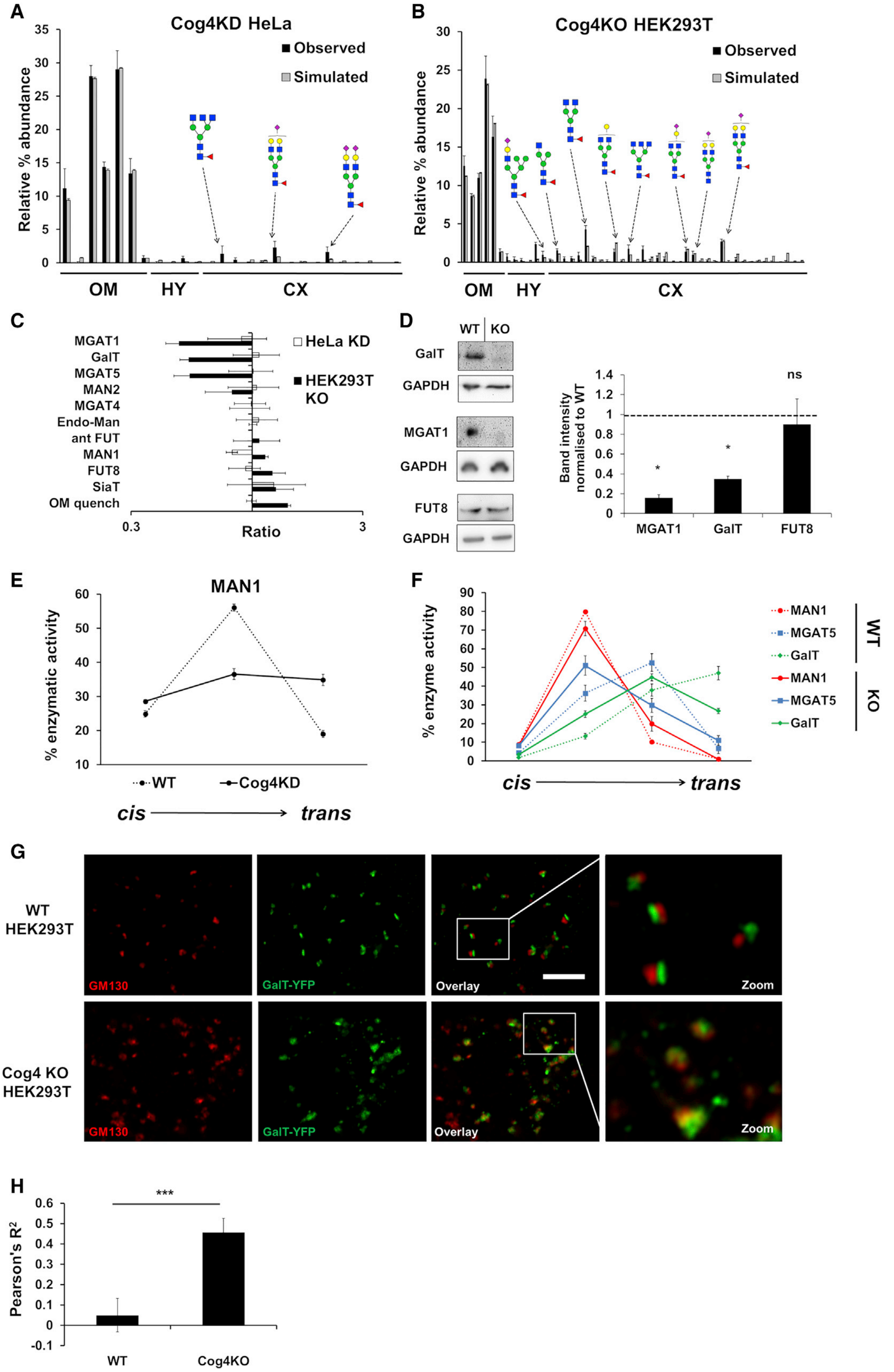

(legend on next page)

matched the drug-treated experimental glycan profile (Figure 3F), demonstrating that the fitting procedure is required to capture novel features in glycan processing.

Predicting the Impact of Trafficking Defects

Several conditions, such as CDGs, cancers, and cellular differentiation, can alter the levels and localizations of glycosylation enzymes. Experimentally determining such changes, in particular altered cisternal localization, is difficult, especially when dealing with a dozen or more enzymes. Perturbing COG subunits alters cellular glycan profiles (Abdul Rahman et al., 2014; Bailey Blackburn et al., 2016; Skeene et al., 2017). Therefore, to test the model's ability to predict changes in enzyme levels and localizations, we fitted the glycan profiles of a Cog4 knockdown (Cog4KD) HeLa cell line and a Cog4 knockout (Cog4KO) HEK293T cell line, in each case starting with the fitted parameter set for the respective wild-type (WT) profile. The altered relative intensities of oligomannose-type glycans in Cog4KD HeLa cells could be replicated *in silico* (Figure 4A). The alteration to the oligomannose abundance leads our model to predict MAN1 distribution to flatten out and shift to a more *trans*-Golgi localization (Figure 4E). Moreover, a reduction in the overall effective MAN1 rate is predicted by the model (Figure 4C).

The Cog4KD HeLa cell model focuses on oligomannose glycans and their modifying enzymes. In contrast, the Cog4KO HEK293T cells show marked changes in complex glycans: they show large decreases in the levels of fucosylation and sialylation, in addition to redistribution of oligomannose glycans (Bailey Blackburn et al., 2016) without a significant change in the cell surface proteome (Figure S6; Table S3). To simulate the glycan profile of these cells (Figure 4B), the model predicts decreases in the total effective enzymatic rates of *N*-acetylglucosaminyltransferase I (MGAT1), *N*-acetylglucosaminyltransferase V (MGAT5), MAN2, and galactosyltransferase (GalT) (Figure 4C; Figure S3). We validated the predicted decreases in GalT and MGAT1 levels through western blotting (Figure 4D). Unexpectedly, the model predicts the effective enzymatic rate of FUT8 to show no significant change upon Cog4KO, despite the observed reduction in overall fucosylation levels (Bailey Blackburn et al., 2016), a prediction also validated by western blotting (Figure 4D).

Upon fitting the Cog4KO HEK293T profile, three enzymes exhibited localization changes compared with WT (Figure 4F). The distribution of MAN1 was shifted in the *trans* direction, although to a smaller degree compared to Cog4KD HeLa cells. In contrast, the proportions of MGAT5 in the third cisterna and GalT in the fourth cisterna were reduced upon Cog4KO, indicating a shift of these enzymes toward the *cis* side of the Golgi (Figure 4F). This suggests that the overall loss of enzyme levels is largely due to loss in the *trans*-Golgi. Confocal microscopy showed increased colocalization between *cis*-Golgi marker GM130 and exogenously expressed GalT-YFP in the Cog4KO cells (Figures 4G and 4H), a finding also reported in the literature (Climer et al., 2018).

It was puzzling that neither the level nor the localization of FUT8 was altered in the Cog4KO model. The action of FUT8 was investigated on flux maps showing all reactions needed for generating the computed glycan profiles of WT and Cog4KO HEK293T cells. The FUT8-catalyzed reactions with the highest proportion of flux (i.e., the most prominent fucosylation steps) cluster away from the most abundant fucosylated glycans (compare blue and red dots in Figure 5A and Figure S4). The main fucosylation reactions occur early during complex *N*-glycan processing, while the most abundant fucosylated glycans are mature complex glycans (Figure 5A; Figure S4). We therefore compared the fluxes of the reactions competing with the top six FUT8 reactions between WT and Cog4KO cells (Figure 5B; Table 1). After weighting these fluxes using the total fucosylation flux in the given cell line, the Cog4KO cell line showed more preference to fucosylate $Hex_4HexNAc_4$ than WT due to less competition by GalT and MGAT5. Overall, the weighted ratios between the two sets of fluxes suggest that the observed decrease in total fucosylation is due to a lower total amount of complex *N*-glycans. However, when comparing WT and knockout (KO) cells, flux distributions are altered in ways that do not always lend themselves to simplistic explanation. For example, $Hex_3HexNAc_4$ is galactosylated more in KO cells at the expense of branching by MGAT5. In contrast, galactosylation of $Hex_4HexNAc_4$ is reduced in KO cells due to sialyltransferase (SiaT) and FUT8, which become dominant over GalT for the $Hex_4HexNAc_4$ substrate. Thus, our model is able to predict unintuitive changes in enzyme homeostasis and fluxes through glycan processing reactions.

Previous work combining computational simulations of the *N*-glycosylation reaction network and *in vitro* experiments in Chinese hamster ovary (CHO) cells has shown that the suppression of GalT can lead to the formation of higher amounts of tri- and tetra-antennary glycans (McDonald et al., 2014). We sought to

Figure 4. Predicting Organizational Changes in Trafficking Defective Cell Lines

(A and B) Observed and simulated glycan profiles of Cog4KD HeLa cell lines (A) and Cog4KO HEK293T cell lines (B). Error bars for glycan profiling are SEM for n = 3.

(C) Total effective enzymatic rate changes predicted by the model following fitting as a result of Cog4 perturbation. The x axis is on a log scale. Error bars for total effective enzymatic rates and distributions are SD for n = 20 (Cog4KD HeLa) and n = 21 (Cog4KO HEK293T) individual fitting procedures.

(D) Western blot analysis of endogenous MGAT1, GalT, and FUT8 and quantification normalized to WT HEK293T. Error bars for western blot quantification are SD for n = 3. ns, not significant; *$p < 0.05$ for a Student's t test.

(E) Predicted relative distribution of MAN1 following the fitting of WT and Cog4KD HeLa glycan profiles.

(F) Predicted relative distributions of selected enzymes in WT and Cog4KO HEK293T cells in each cisterna normalized to the total predicted effective enzymatic rate for each enzyme for each cell line.

(G) Airyscan confocal microscopy of GM130 and exogenous GalT-YFP in nocodazole-treated WT and Cog4KO HEK293T cell lines.

(H) Pearson's correlation coefficient for WT and Cog4KO HEK293T cells. Pearson's correlation coefficients were calculated for each Golgi stack, and error bars are SD for n = 4 cells with 151 (WT) and 131 (Cog4KO) stacks. ***$p < 0.001$ for a Student's t test.

See also Figures S3 and S6 and Table S3.

OPEN
ACCESS
CellPress

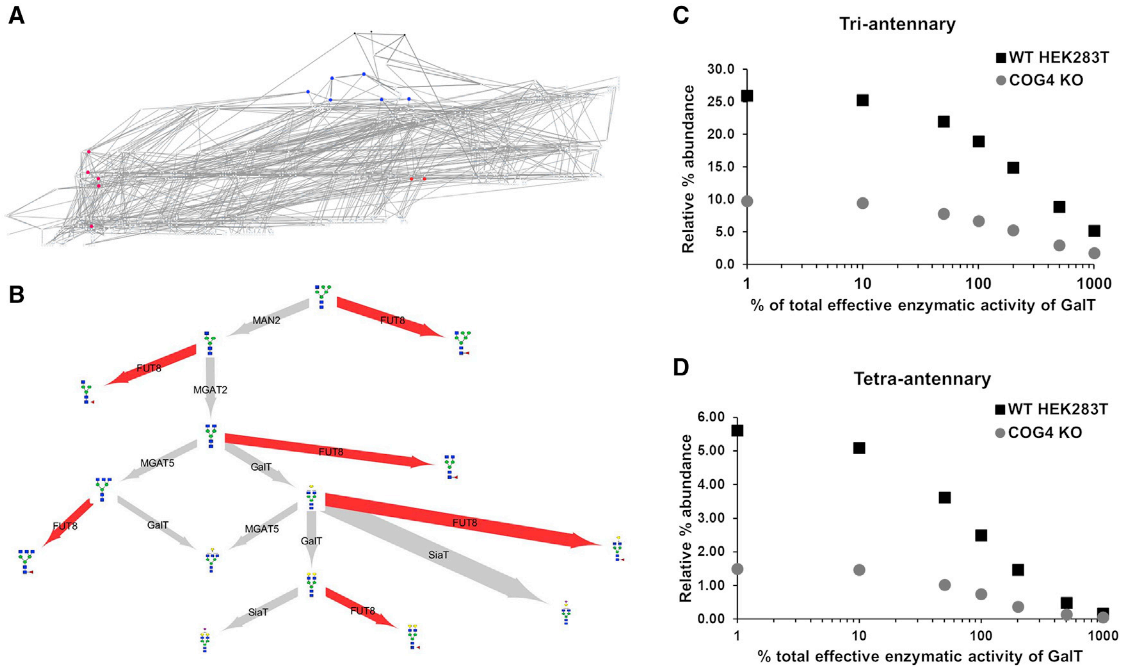

Figure 5. Investigating Glycan Flux in HEK293T Cells

(A) Total flux map for all enzymatic glycan processing reactions for 10,000 input glycans occurring during the simulation of the WT HEK293T glycan profile. Blue dots represent the substrates of the six reactions with the highest fluxes of all FUT8-catalyzed reactions. Red dots represent the most abundant fucosylated glycans in the observed glycan profile of these cells.

(B) Weighted ratio of the Cog4KO flux to the WT flux for the top six substrates for FUT8. Red arrows highlight the fluxes of the FUT8 enzyme. Arrow thickness denotes the ratio of flux for Cog4KO and WT HEK293T cells divided by the ratio of the total core fucosylation flux for Cog4KO and WT HEK293T cells.

(C and D) Simulated relative percent abundance of tri-antennary glycans (C) and tetra-antennary glycans (D) as it varies with the total effective enzymatic rate of GalT in WT (black squares) and Cog4KO (gray circles) HEK293T cells. The percent total effective enzymatic rate of GalT is plotted on a log scale.

See also Figure S4.

test whether this effect is also produced using our stochastic model of glycosylation in WT and Cog4KO HEK293T cells. In agreement with previous work (McDonald et al., 2014), varying the effective enzymatic activity of only GalT can control glycan branching. Increasing GalT activity decreased the abundance of both tri- and tetra-antennary glycans as reported (Figures 5C and 5D) (McDonald et al., 2014). The maximum relative abundance of highly branched glycans that could be reached at low GalT activities was considerably lower for the Cog4KO cells compared to WT cells.

MSCs

Glycosylation has been shown to affect MSC differentiation potential (Wilson et al., 2018). To investigate the changes in *N*-glycan biosynthesis during differentiation, we modeled glycan profiles of Y101 MSCs and their derived osteoblasts. To serve as a starting point for fitting of the osteoblast profile, the glycan profile of Y101 MSCs was modeled (Figure 6A; Table S6), starting with the WT HEK293T parameters. Due to the used scoring system's reliance on the square of the difference between the simulated and the observed relative glycan abundance, it was possible to improve the fit by manually changing enzyme parameters without altering the overall score. Such manual fine-tuning, which was used for the final fitting of the Y101 MSC model, avoided the need to introduce multiple scoring systems with different sensitivities to different features of the model. We stress that manual fine-tuning of the parameters was only undertaken when we did not wish to directly compare the two cell line models (e.g., WT HEK293T to Y101 MSCs).

The glycan profile of osteoblasts (Wilson et al., 2016) was then fitted (Figure 6B; Table S7) starting from the Y101 MSC model to reveal changes to the glycosylation machinery that occur during osteogenic differentiation. Osteogenic differentiation results in an increased complex-to-oligomannose glycan ratio, causing the model to reduce the rate of OM quenching. The recycling of ER glycoproteins was not expected to decrease significantly during osteogenesis. This would suggest decreased lysosomal content in osteoblasts compared to MSCs, yet we found the opposite (Figure S5) (Nabavi et al., 2008; Taniguchi et al., 2010). The increased amount of complex glycan observed upon osteogenesis is the main cause for the reduced OM quench effective rate. However, this shift to more complex glycans could be due to the differentiating cells cumulatively laying down the complex *N*-glycan-containing extracellular matrix while turning over intracellular (including lysosomal and ER) glycoproteins. Hence, we focused on predictions of the model pertaining to complex glycans only. MGAT5 levels are predicted to decrease and move away from medial cisternae in

Table 1. WT and Cog4 KO Fluxes

Glycan Source	Normalized KO/WT	Glycan Target	Enzyme
$Hex_3HexNAc_3$	0.83	$Fuc_1Hex_3HexNAc_3$	FUT8
$Hex_3HexNAc_3$	0.67	$Hex_3HexNAc_4$	MGAT2
$Hex_3HexNAc_4$	0.79	$Hex_4HexNAc_4$	GalT
$Hex_3HexNAc_4$	0.81	$Fuc_1Hex_3HexNAc_4$	FUT8
$Hex_3HexNAc_4$	0.37	$Hex_3HexNAc_5$	MGAT5
$Hex_3HexNAc_5$	0.49	$Fuc_1Hex_3HexNAc_5$	FUT8
$Hex_3HexNAc_5$	0.17	$Hex_4HexNAc_5$	GalT
$Hex_4HexNAc_4$	0.82	$Hex_5HexNAc_4$	GalT
$Hex_4HexNAc_4$	1.53	$Fuc_1Hex_4HexNAc_4$	FUT8
$Hex_4HexNAc_4$	2.64	$NeuAc_1Hex_4HexNAc_4$	SiaT
$Hex_4HexNAc_4$	0.31	$Hex_4HexNAc_5$	MGAT5
$Hex_5HexNAc_3$	0.97	$Fuc_1Hex_5HexNAc_3$	FUT8
$Hex_5HexNAc_3$	0.71	$Hex_3HexNAc_3$	MAN2
$Hex_5HexNAc_4$	0.60	$Fuc_1Hex_5HexNAc_4$	FUT8
$Hex_5HexNAc_4$	0.61	$NeuAc_1Hex_5HexNAc_4$	SiaT

Normalized flux between glycan substrate and glycan product. The normalization is calculated by taking the ratio of flux between each substrate and product for Cog4KO and WT HEK293T cells and multiplying by the ratio of the total core fucosylation flux for Cog4KO and WT HEK293T cells. Normalized KO/WT fluxes over 1.0 reflect a preference for this pathway in the Cog4KO HEK293T cell line, and vice versa.

osteoblasts (Figures 6C and 6D). This correlates with a noticeable shift from tri- to bi-antennary glycans in osteoblasts compared to MSCs. Altered branching affects galectin binding (Demetriou et al., 2001). Galectins in turn have been shown to prevent the differentiation of pre-osteoblasts (Nakajima et al., 2014). We hypothesized that a similar mechanism could operate earlier than the pre-osteoblast stage in the differentiation process, because inhibiting complex glycan formation was shown to enhance the initial commitment of MSCs to osteogenic differentiation (Wilson et al., 2018). Inhibiting galectin binding with lactose during the first 7 days of a 21-day osteogenesis program increased osteogenic differentiation (Figures 6E and 6F). This implies that the reduction in *N*-glycan antenna number, which is observed in osteoblasts and predicted to be a consequence of altered MGAT5 levels and localizations, is a contributing factor in osteogenic differentiation.

DISCUSSION

This work has generated a computational model of Golgi-based *N*-glycan processing through iteration of computation and experiments. By using our *in silico* methodology to reproduce the qualitative features, as well as to create high-quality quantitative fits for glycan profiles, we can predict alterations in the enzyme organization of cell lines that result from disruptions to the Golgi trafficking machinery (Bailey Blackburn et al., 2016).

Nonetheless, modeling needs to be seen as a process of discovery, rather than simply an end result. As the cycles of iterative modeling progressed from HeLa cells to describe the more intricate HEK293T and MSC glycan profiles, it became clear that substrate specificity needed to be included for several enzymes. Our fitting suggested the profiles were particularly sensitive to GalT and SiaT, so substrate specificity was selectively introduced by applying scaling factors that are consistent with the substrate specificities of enzymes reported in the literature (Barb et al., 2009; Joziasse et al., 1987; Ramasamy et al., 2005). Our results also suggest that variable substrate specificity could be particularly relevant for hybrid glycans that were often poorly modeled when compared with complex glycans. Only a small number of differences were found between cell surface proteomes of WT and those of Cog4KO HEK293T cells (Figure S6), suggesting that alterations in the glycan profile due to site-specific glycosylation (Losfeld et al., 2017) are likely to be limited. However, we cannot eliminate the influence of site-specific glycosylation on changes in the osteoblast glycan profile. To improve the accuracy of modeling in the future, the variability in the half-lives of subsets of glycoproteins could also be considered.

Our primary goal in developing our modeling technique was to understand the perturbations of the glycosylation enzymes caused by defects in the COG complex. Defects in this trafficking complex cause several CDGs by altering the recycling, and consequently the levels and localizations, of glycosylation enzymes (Oka et al., 2004; Steet and Kornfeld, 2006). For the comparison of cell lines with a disrupted COG complex (e.g., WT HEK293T versus Cog4KO HEK293), we made the assumption that the enzymatic rate constants do not change between the cell lines we compared. This is because disruption to the COG complex alters enzyme sorting, not the physical state of the enzyme. Therefore, in these COG mutant cases, predictions of the changes in enzyme activity parameters represent changes in the amount of enzyme available within specific cisternae. This is the main prediction on which we have focused our validations.

Our model provides an excellent tool to predict all alterations to the effective enzymatic rates of the glycosylation enzymes and their localizations, and thereby shed light on glycan biosynthetic details in Golgi trafficking-defective cell lines. Modeling the Cog4KD HeLa cell profile has led us to predict a shift in the *trans* direction of the MAN1 enzyme. Because complex glycan levels are low, we can only make confident predictions regarding the enzymes acting on oligomannose glycans in this system. In contrast, modeling the glycan profile of Cog4KO HEK293T cells predicted shifts in the levels and/or localizations of several enzymes acting on complex glycans. This included decreased levels and a shift toward the *cis*-Golgi for GalT and MGAT5 as a result of Cog4KO. Other enzyme levels, such as those of FUT8, are predicted to be less sensitive to Cog4KO, and several of these predictions, including the altered GalT localization, agree with previous reports (Climer et al., 2018) and/or were validated experimentally.

The model allowed us to generate flux maps describing the variation in glycan biosynthetic pathways as a consequence of the mutation. This information highlights which reactions could be best targeted to correct the glycan profile of a patient or to glyco-engineer recombinant therapeutic proteins. For example, by isolating the most abundant fucosylation reactions *in silico*, we found that $Hex_4HexNAc_4$ was a more favorable substrate for

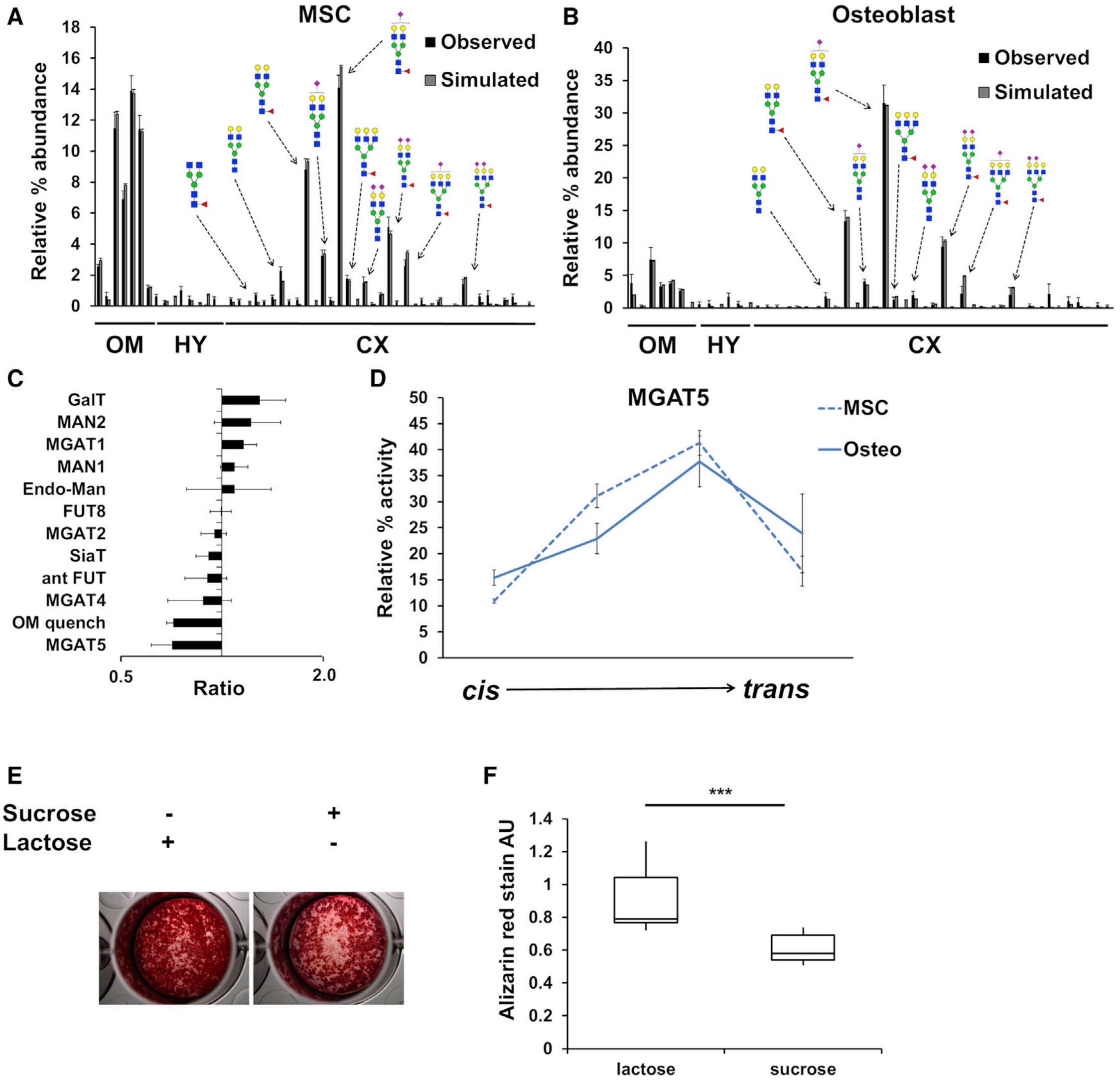

Figure 6. Predicting Alterations in Glycan Processing as a Result of Osteogenesis

(A and B) Observed and simulated glycan profiles of MSCs (A) and MSCs following 21-day osteogenic differentiation (B).

(C) Total effective enzymatic rate changes predicted to occur upon differentiation derived following fitting of the osteoblast glycan profile. The x axis is shown on a log scale.

(D) Distribution of MGAT5 in the Golgi stack, as predicted by the fitting of Y101 MSC and osteoblast glycan profiles. Error bars for glycan profiling are SEM for n = 3. Error bars for total effective enzymatic rates and distributions are SD for n = 14 (MSC) and n = 20 (osteoblast) individual fitting runs.

(E) Representative alizarin red-stained Y101 MSCs following 21-day osteogenic differentiation in the presence of either sucrose or lactose during the first 7 days.

(F) Quantification of the eluted alizarin red stain for n = 10. ***$p < 0.001$.

See also Figure S5 and Tables S6 and S7.

FUT8 in the Cog4KO cells than in WT. This was a consequence of less competition from GalT and MGAT5 for this substrate. Thus, although decreased fucosylation flux in the Cog4KO cells is due to lower complex glycan levels (a consequence of reduced MGAT1), simple overexpression of MGAT1 would result in increased fucosylation but with an altered profile compared to WT. This makes our model a potentially powerful tool in the development of treatments for COG-CDGs (Wu et al., 2004).

We also used our modeling methodology to predict adaptions of the glycosylation machinery during the differentiation of MSCs into osteoblasts. We observed an MGAT5-mediated shift from tri- to bi-antennary glycans upon differentiation. The degree of *N*-glycan branching has been linked to receptor endocytosis that affects cell proliferation and differentiation (Lau et al., 2007) via galectin binding (Demetriou et al., 2001). Inhibition of galectin binding by lactose promoted osteogenesis even earlier during differentiation in our hands than previously reported (Nakajima et al., 2014). Lactose was effective in the first week of the 3-week process. This is similar in timing to kifunensine, a drug that abrogates complex branching, and promoted differentiation

within the first week (Wilson et al., 2018). This suggests that a switch from tri- to bi-antennary glycans releases an inhibitory effect on differentiation, likely by promoting the endocytosis of a key receptor or receptors (Lau et al., 2007).

In summary, we have developed a computational workflow to predict the architecture of the glycan biosynthetic machinery in the Golgi based on an SSA and an approximate Bayesian computation (ABC) fitting algorithm. Although other fitting methodologies, such as genetic algorithms (Liu et al., 2008), a non-linear least-squares (Marquardt-Levenberg) method (Krambeck et al., 2017), and maximum likelihood estimation (Sou et al., 2017), have been used in glycosylation modeling, our use of a Bayesian fitting approach allows us to use prior knowledge of the system to suggest solutions most compatible with both data and existing knowledge. Through the use of Bayesian fitting, we can make comparisons between cell lines in a high-dimensional parameter space with uncertain quality of information. This enables us to make progress, especially when understanding changes among biological settings. We show that our methodology is capable of generating accurate and testable hypotheses regarding enzyme levels and localizations by simulating the glycan profiles of human cell lines perturbed through drug treatments, genetic manipulation, and even a process as complex as differentiation. Applying our approach to Golgi trafficking-defective cell lines allowed us to predict which enzymes may be sensitive to Cog4KO, thus presenting an *in silico* approach to the experimental identification of COG-sensitive Golgi proteins (Oka et al., 2004). The ability to predict alterations to Golgi enzymes, as well as to probe *in silico*-generated flux maps to identify changes in glycosylation pathways, has possible applications in the understanding and treatment of COG-CDGs and the potential to be used in the glyco-engineering of biologics.

STAR★METHODS

Detailed methods are provided in the online version of this paper and include the following:

- KEY RESOURCES TABLE
- CONTACT FOR REAGENT AND RESOURCE SHARING
- EXPERIMENTAL MODEL AND SUBJECT DETAILS
- METHOD DETAILS
  - Cell culture and osteogenic differentiation
  - Modeling framework
  - Stochastic simulation algorithm
  - Approximate Bayesian computation without likelihood (*cf.* Figure S1)
  - Confocal Microscopy
  - Alizarin red staining
  - Western blotting
  - *N*-glycan release
  - Permethylation
  - Glycan mass spectrometry
  - Crude membrane proteomics
  - Beta-hexosaminidase assay
- QUANTIFICATION AND STATISTICAL ANALYSIS
- DATA AND SOFTWARE AVAILABILITY

SUPPLEMENTAL INFORMATION

Supplemental Information can be found online at https://doi.org/10.1016/j.celrep.2019.03.107.

ACKNOWLEDGMENTS

We gratefully acknowledge cell lines and critical advice from Vladimir Lupashin (UAMS, Little Rock) and Paul Genever (University of York). We are grateful to the York Bioscience Technology Facility Metabolomics and Proteomics lab, in particular Adam Dowle, for expert help with glycomic and proteomic analysis. The Imaging Facility at the York Bioscience Technology Facility helped with confocal microscopy analysis, and the York Research Computing Cluster was used for the computational modeling work. All mass spectrometric analyses used instruments in the York Centre of Excellence in Mass Spectrometry, which was created by a major capital investment through Science City York, supported by Yorkshire Forward with funds from the Northern Way Initiative, and subsequent support from EPSRC (EP/K039660/1 and EP/M028127/1). We thank Nia Bryant, Neil Bruce (University of York), Rob Field (John Innes Centre), Fred Hughson (Princeton University), Martin Lowe (University of Manchester), and Vladimir Lupashin (UAMS, Little Rock) for critical comments on the manuscript. This work was supported by a BBSRC IB Catalyst grant (BB/M018237/1). P.F. was funded by the BBSRC White Rose DTP (BB/J014443/1) studentship awarded to D.U., A.J.W., and J.T.-O. H.S. was funded by a BBSRC NPIF iCASE studentship awarded to D.U. and J.T.-O. (BB/R506382/1).

AUTHOR CONTRIBUTIONS

D.U., A.J.W., J.T.-O., and P.F. designed the study. P.F., D.U., A.J.W., and J.T-.O. planned the experiments, which were performed by P.F. and H.S. A.J.W. and P.F. wrote the modeling software, and P.F. conducted the computational modeling. P.F., D.U., A.J.W., and J.T.-O. wrote the manuscript. D.U. coordinated the project.

DECLARATION OF INTERESTS

The authors declare no competing interests.

Received: October 10, 2018
Revised: January 24, 2019
Accepted: March 27, 2019
Published: April 23, 2019

REFERENCES

Abdul Rahman, S., Bergström, E., Watson, C.J., Wilson, K.M., Ashford, D.A., Thomas, J.R., Ungar, D., and Thomas-Oates, J.E. (2014). Filter-aided *N*-glycan separation (FANGS): a convenient sample preparation method for mass spectrometric *N*-glycan profiling. J. Proteome Res. *13*, 1167–1176.

Bailey Blackburn, J., Pokrovskaya, I., Fisher, P., Ungar, D., and Lupashin, V.V. (2016). COG Complex Complexities: Detailed Characterization of a Complete Set of HEK293T Cells Lacking Individual COG Subunits. Front. Cell Dev. Biol. *4*, 23.

Barb, A.W., Brady, E.K., and Prestegard, J.H. (2009). Branch-specific sialylation of IgG-Fc glycans by ST6Gal-I. Biochemistry *48*, 9705–9707.

Bause, E., Breuer, W., Schweden, J., Roeser, R., and Geyer, R. (1992). Effect of substrate structure on the activity of Man9-mannosidase from pig liver involved in *N*-linked oligosaccharide processing. Eur. J. Biochem. *208*, 451–457.

Belloni, G., Sechi, S., Riparbelli, M.G., Fuller, M.T., Callaini, G., and Giansanti, M.G. (2012). Mutations in Cog7 affect Golgi structure, meiotic cytokinesis and sperm development during *Drosophila* spermatogenesis. J. Cell Sci. *125*, 5441–5452.

Bieberich, E. (2014). Synthesis, Processing, and Function of *N*-glycans in *N*-glycoproteins. Adv. Neurobiol. *9*, 47–70.

OPEN ACCESS
CellPress

Christie, D.R., Shaikh, F.M., Lucas, J.A., 4th, Lucas, J.A., 3rd, and Bellis, S.L. (2008). ST6Gal-I expression in ovarian cancer cells promotes an invasive phenotype by altering integrin glycosylation and function. J. Ovarian Res. *1*, 3.

Climer, L.K., Pokrovskaya, I.D., Blackburn, J.B., and Lupashin, V.V. (2018). Membrane detachment is not essential for COG complex function. Mol. Biol. Cell *29*, 964–974. PubMed.

Cottam, N.P., Wilson, K.M., Ng, B.G., Körner, C., Freeze, H.H., and Ungar, D. (2014). Dissecting functions of the conserved oligomeric Golgi tethering complex using a cell-free assay. Traffic *15*, 12–21.

Crispin, M., Harvey, D.J., Chang, V.T., Yu, C., Aricescu, A.R., Jones, E.Y., Davis, S.J., Dwek, R.A., and Rudd, P.M. (2006). Inhibition of hybrid- and complex-type glycosylation reveals the presence of the GlcNAc transferase I-independent fucosylation pathway. Glycobiology *16*, 748–756.

Csilléry, K., Blum, M.G.B., Gaggiotti, O.E., and François, O. (2010). Approximate Bayesian Computation (ABC) in practice. Trends Ecol. Evol. *25*, 410–418.

Demetriou, M., Granovsky, M., Quaggin, S., and Dennis, J.W. (2001). Negative regulation of T-cell activation and autoimmunity by Mgat5 *N*-glycosylation. Nature *409*, 733–739.

Doob, J.L. (1945). Markoff Chains–Denumerable Case. Trans. Am. Math. Soc. *58*, 455.

Dunphy, W.G., and Rothman, J.E. (1983). Compartmentation of asparagine-linked oligosaccharide processing in the Golgi apparatus. J. Cell Biol. *97*, 270–275.

Elbein, A.D., Solf, R., Dorling, P.R., and Vosbeck, K. (1981). Swainsonine: an inhibitor of glycoprotein processing. Proc. Natl. Acad. Sci. USA *78*, 7393–7397.

Eldar, A., and Elowitz, M.B. (2010). Functional roles for noise in genetic circuits. Nature *467*, 167–173.

Ferrara, C., Brünker, P., Suter, T., Moser, S., Püntener, U., and Umaña, P. (2006). Modulation of therapeutic antibody effector functions by glycosylation engineering: influence of Golgi enzyme localization domain and co-expression of heterologous β1, 4-*N*-acetylglucosaminyltransferase III and Golgi α-mannosidase II. Biotechnol. Bioeng. *93*, 851–861.

Fisher, P., and Ungar, D. (2016). Bridging the Gap between Glycosylation and Vesicle Traffic. Front. Cell Dev. Biol. *4*, 15.

Fukuda, M., Kanno, E., Ishibashi, K., and Itoh, T. (2008). Large scale screening for novel rab effectors reveals unexpected broad Rab binding specificity. Mol. Cell. Proteomics *7*, 1031–1042.

Gelman, A., and Rubin, D.B. (1992). Inference from Iterative Simulation Using Multiple Sequences. Stat. Sci. *7*, 457–472.

Gibson, M.A., and Bruck, J. (2000). Efficient Exact Stochastic Simulation of Chemical Systems with Many Species and Many Channels. J. Phys. Chem. A *104*, 1876–1889.

Gillespie, D.T. (1976). A general method for numerically simulating the stochastic time evolution of coupled chemical reactions. J. Comp. Physiol. *22*, 403–434.

Harris, S.L., and Waters, M.G. (1996). Localization of a yeast early Golgi mannosyltransferase, Och1p, involves retrograde transport. J. Cell Biol. *132*, 985–998.

Hemmerich, S., Bertozzi, C.R., Leffler, H., and Rosen, S.D. (1994). Identification of the sulfated monosaccharides of GlyCAM-1, an endothelial-derived ligand for L-selectin. Biochemistry *33*, 4820–4829.

Hossler, P., Mulukutla, B.C., and Hu, W.-S. (2007). Systems analysis of *N*-glycan processing in mammalian cells. PLoS ONE *2*, e713.

James, S., Fox, J., Afsari, F., Lee, J., Clough, S., Knight, C., Ashmore, J., Ashton, P., Preham, O., Hoogduijn, M., et al. (2015). Multiparameter Analysis of Human Bone Marrow Stromal Cells Identifies Distinct Immunomodulatory and Differentiation-Competent Subtypes. Stem Cell Reports *4*, 1004–1015.

Joziasse, D.H., Schiphorst, W.E., Van den Eijnden, D.H., Van Kuik, J.A., Van Halbeek, H., and Vliegenthart, J.F. (1987). Branch specificity of bovine colostrum CMP-sialic acid: Gal beta 1——4GlcNAc-R alpha 2——6-sialyltransferase. Sialylation of bi-, tri-, and tetraantennary oligosaccharides and glycopeptides of the *N*-acetyllactosamine type. J. Biol. Chem. *262*, 2025–2033.

Kodera, H., Ando, N., Yuasa, I., Wada, Y., Tsurusaki, Y., Nakashima, M., Miyake, N., Saitoh, S., Matsumoto, N., and Saitsu, H. (2015). Mutations in COG2 encoding a subunit of the conserved oligomeric golgi complex cause a congenital disorder of glycosylation. Clin. Genet. *87*, 455–460.

Krambeck, F.J., and Betenbaugh, M.J. (2005). A mathematical model of *N*-linked glycosylation. Biotechnol. Bioeng. *92*, 711–728.

Krambeck, F.J., Bennun, S.V., Narang, S., Choi, S., Yarema, K.J., and Betenbaugh, M.J. (2009). A mathematical model to derive *N*-glycan structures and cellular enzyme activities from mass spectrometric data. Glycobiology *19*, 1163–1175.

Krambeck, F.J., Bennun, S.V., Andersen, M.R., and Betenbaugh, M.J. (2017). Model-based analysis of *N*-glycosylation in Chinese hamster ovary cells. PLoS ONE *12*, e0175376.

Lal, A., Pang, P., Kalelkar, S., Romero, P.A., Herscovics, A., and Moremen, K.W. (1998). Substrate specificities of recombinant murine Golgi alpha1, 2-mannosidases IA and IB and comparison with endoplasmic reticulum and Golgi processing alpha1,2-mannosidases. Glycobiology *8*, 981–995.

Lau, K.S., Partridge, E.A., Grigorian, A., Silvescu, C.I., Reinhold, V.N., Demetriou, M., and Dennis, J.W. (2007). Complex *N*-glycan number and degree of branching cooperate to regulate cell proliferation and differentiation. Cell *129*, 123–134.

Lin, A.I., Philipsberg, G.A., and Haltiwanger, R.S. (1994). Core fucosylation of high-mannose-type oligosaccharides in GlcNAc transferase I-deficient (Lec1) CHO cells. Glycobiology *4*, 895–901.

Liu, G., Marathe, D.D., Matta, K.L., and Neelamegham, S. (2008). Systems-level modeling of cellular glycosylation reaction networks: O-linked glycan formation on natural selectin ligands. Bioinformatics *24*, 2740–2747.

Losfeld, M.-E., Scibona, E., Lin, C.-W., Villiger, T.K., Gauss, R., Morbidelli, M., and Aebi, M. (2017). Influence of protein/glycan interaction on site-specific glycan heterogeneity. FASEB J. *31*, 4623–4635.

Marjoram, P., Molitor, J., Plagnol, V., and Tavare, S. (2003). Markov chain Monte Carlo without likelihoods. Proc. Natl. Acad. Sci. USA *100*, 15324–15328.

Marra, P., Maffucci, T., Daniele, T., Tullio, G.D., Ikehara, Y., Chan, E.K., Luini, A., Beznoussenko, G., Mironov, A., and De Matteis, M.A. (2001). The GM130 and GRASP65 Golgi proteins cycle through and define a subdomain of the intermediate compartment. Nat. Cell Biol. *3*, 1101–1113.

McDonald, A.G., Hayes, J.M., Bezak, T., Głuchowska, S.A., Cosgrave, E.F.J., Struwe, W.B., Stroop, C.J.M., Kok, H., van de Laar, T., Rudd, P.M., et al. (2014). Galactosyltransferase 4 is a major control point for glycan branching in *N*-linked glycosylation. J. Cell Sci. *127*, 5014–5026.

Mehta, N., Porterfield, M., Struwe, W.B., Heiss, C., Azadi, P., Rudd, P.M., Tiemeyer, M., and Aoki, K. (2016). Mass Spectrometric Quantification of *N*-Linked Glycans by Reference to Exogenous Standards. J. Proteome Res. *15*, 2969–2980.

Miller, V.J., Sharma, P., Kudlyk, T.A., Frost, L., Rofe, A.P., Watson, I.J., Duden, R., Lowe, M., Lupashin, V.V., and Ungar, D. (2013). Molecular insights into vesicle tethering at the Golgi by the conserved oligomeric Golgi (COG) complex and the golgin TATA element modulatory factor (TMF). J. Biol. Chem. *288*, 4229–4240.

Nabavi, N., Urukova, Y., Cardelli, M., Aubin, J.E., and Harrison, R.E. (2008). Lysosome dispersion in osteoblasts accommodates enhanced collagen production during differentiation. J. Biol. Chem. *283*, 19678–19690.

Nakajima, K., Kho, D.H., Yanagawa, T., Harazono, Y., Gao, X., Hogan, V., and Raz, A. (2014). Galectin-3 inhibits osteoblast differentiation through notch signaling. Neoplasia *16*, 939–949.

Oka, T., Ungar, D., Hughson, F.M., and Krieger, M. (2004). The COG and COPI complexes interact to control the abundance of GEARs, a subset of Golgi integral membrane proteins. Mol. Biol. Cell *15*, 2423–2435.

Orlando, R. (2010). Quantitative glycomics. Methods Mol. Biol. *600*, 31–49. PubMed.

Pokrovskaya, I.D., Willett, R., Smith, R.D., Morelle, W., Kudlyk, T., and Lupashin, V.V. (2011). Conserved oligomeric Golgi complex specifically regulates the maintenance of Golgi glycosylation machinery. Glycobiology *21*, 1554–1569.

Rabouille, C., Hui, N., Hunte, F., Kieckbusch, R., Berger, E.G., Warren, G., and Nilsson, T. (1995). Mapping the distribution of Golgi enzymes involved in the construction of complex oligosaccharides. J. Cell Sci. *108* (*Pt 4*), 1617–1627. PubMed.

Ramasamy, V., Ramakrishnan, B., Boeggeman, E., Ratner, D.M., Seeberger, P.H., and Qasba, P.K. (2005). Oligosaccharide preferences of β1,4-galactosyltransferase-I: crystal structures of Met340His mutant of human β1,4-galactosyltransferase-I with a pentasaccharide and trisaccharides of the *N*-glycan moiety. J. Mol. Biol. *353*, 53–67.

Scott, H., and Panin, V.M. (2014). The role of protein *N*-glycosylation in neural transmission. Glycobiology *24*, 407–417.

Sherlock, C., Thiery, A.H., Roberts, G.O., and Rosenthal, J.S. (2015). On the efficiency of pseudo-marginal random walk Metropolis algorithms. Ann. Statist. *43*, 238–275.

Shields, R.L., Lai, J., Keck, R., O'Connell, L.Y., Hong, K., Meng, Y.G., Weikert, S.H.A., and Presta, L.G. (2002). Lack of fucose on human IgG1 *N*-linked oligosaccharide improves binding to human Fcgamma RIII and antibody-dependent cellular toxicity. J. Biol. Chem. *277*, 26733–26740.

Skeene, K., Walker, M., Clarke, G., Bergström, E., Genever, P., Ungar, D., and Thomas-Oates, J. (2017). One Filter, One Sample, and the *N*- and *O*-Glyco (proteo)me: Toward a System to Study Disorders of Protein Glycosylation. Anal. Chem. *89*, 5840–5849.

Sou, S.N., Jedrzejewski, P.M., Lee, K., Sellick, C., Polizzi, K.M., and Kontoravdi, C. (2017). Model-based investigation of intracellular processes determining antibody Fc-glycosylation under mild hypothermia. Biotechnol. Bioeng. *114*, 1570–1582.

Spahn, P.N., Hansen, A.H., Hansen, H.G., Arnsdorf, J., Kildegaard, H.F., and Lewis, N.E. (2016). A Markov chain model for *N*-linked protein glycosylation—towards a low-parameter tool for model-driven glycoengineering. Metab. Eng. *33*, 52–66.

Stanley, P., and Sudo, T. (1981). Microheterogeneity among carbohydrate structures at the cell surface may be important in recognition phenomena. Cell *23*, 763–769.

Steet, R., and Kornfeld, S. (2006). COG-7-deficient Human Fibroblasts Exhibit Altered Recycling of Golgi Proteins. Mol. Biol. Cell *17*, 2312–2321.

Struwe, W.B., and Reinhold, V.N. (2012). The conserved oligomeric Golgi complex is required for fucosylation of *N*-glycans in *Caenorhabditis elegans*. Glycobiology *22*, 863–875.

Taniguchi, T., Kido, S., Yamauchi, E., Abe, M., Matsumoto, T., and Taniguchi, H. (2010). Induction of endosomal/lysosomal pathways in differentiating osteoblasts as revealed by combined proteomic and transcriptomic analyses. FEBS Lett. *584*, 3969–3974.

Toni, T., and Stumpf, M.P.H. (2010). Simulation-based model selection for dynamical systems in systems and population biology. Bioinformatics *26*, 104–110.

Umaña, P., and Bailey, J.E. (1997). A mathematical model of *N*-linked glycoform biosynthesis. Biotechnol. Bioeng. *55*, 890–908.

Velasco, A., Hendricks, L., Moremen, K.W., Tulsiani, D.R., Touster, O., and Farquhar, M.G. (1993). Cell type-dependent variations in the subcellular distribution of alpha-mannosidase I and II. J. Cell Biol. *122*, 39–51.

Wada, Y., Azadi, P., Costello, C.E., Dell, A., Dwek, R.A., Geyer, H., Geyer, R., Kakehi, K., Karlsson, N.G., Kato, K., et al. (2007). Comparison of the methods for profiling glycoprotein glycans—HUPO Human Disease Glycomics/Proteome Initiative multi-institutional study. Glycobiology *17*, 411–422.

Waetzig, G.H., Chalaris, A., Rosenstiel, P., Suthaus, J., Holland, C., Karl, N., Vallés Uriarte, L., Till, A., Scheller, J., Grötzinger, J., et al. (2010). *N*-linked glycosylation is essential for the stability but not the signaling function of the interleukin-6 signal transducer glycoprotein 130. J. Biol. Chem. *285*, 1781–1789.

Whyte, J.R., and Munro, S. (2001). The Sec34/35 Golgi transport complex is related to the exocyst, defining a family of complexes involved in multiple steps of membrane traffic. Dev. Cell *1*, 527–537.

Willett, R., Ungar, D., and Lupashin, V. (2013). The Golgi puppet master: COG complex at center stage of membrane trafficking interactions. Histochem. Cell Biol. *140*, 271–283.

Willett, R., Blackburn, J.B., Climer, L., Pokrovskaya, I., Kudlyk, T., Wang, W., and Lupashin, V. (2016). COG lobe B sub-complex engages v-SNARE GS15 and functions via regulated interaction with lobe A sub-complex. Sci. Rep. *6*, 29139.

Wilson, K.M., Thomas-Oates, J.E., Genever, P.G., and Ungar, D. (2016). Glycan Profiling Shows Unvaried *N*-Glycomes in MSC Clones with Distinct Differentiation Potentials. Front. Cell Dev. Biol. *4*, 52.

Wilson, K.M., Jagger, A.M., Walker, M., Seinkmane, E., Fox, J.M., Kröger, R., Genever, P., and Ungar, D. (2018). Glycans modify mesenchymal stem cell differentiation to impact the function of resulting osteoblasts. J. Cell Sci. *131*, jcs209452. PubMed.

Wu, X., Steet, R.A., Bohorov, O., Bakker, J., Newell, J., Krieger, M., Spaapen, L., Kornfeld, S., and Freeze, H.H. (2004). Mutation of the COG complex subunit gene COG7 causes a lethal congenital disorder. Nat. Med. *10*, 518–523.

Yang, Q., and Wang, L.X. (2016). Mammalian α1,6-fucosyltransferase (FUT8) is the sole enzyme responsible for the *N*-acetylglucosaminyltransferase I-independent core fucosylation of high-mannose *N*-glycans. J. Biol. Chem. *291*, 11064–11071. PubMed.

Yang, Q., Zhang, R., Cai, H., and Wang, L.X. (2017). Revisiting the substrate specificity of mammalian α1,6-fucosyltransferase (FUT8) reveals that it catalyzes core fucosylation of *N*-glycans lacking α1,3-arm GlcNAc. J. Biol. Chem. *292*, 14796–14803. PubMed.

Zeevaert, R., Foulquier, F., Jaeken, J., and Matthijs, G. (2008). Deficiencies in subunits of the Conserved Oligomeric Golgi (COG) complex define a novel group of Congenital Disorders of Glycosylation. Mol. Genet. Metab. *93*, 15–21.

Zhao, H., Liang, Y., Xu, Z., Wang, L., Zhou, F., Li, Z., Jin, J., Yang, Y., Fang, Z., Hu, Y., et al. (2008). *N*-glycosylation affects the adhesive function of E-Cadherin through modifying the composition of adherens junctions (AJs) in human breast carcinoma cell line MDA-MB-435. J. Cell. Biochem. *104*, 162–175.

STAR★METHODS

KEY RESOURCES TABLE

REAGENT or RESOURCE	SOURCE	IDENTIFIER
Antibodies		
anti-GM130-Alexa-647 (mouse)	BD Biosciences	Clone 35/GM130, 558712
anti-Mgat1 (rabbit)	Abcam	ab180578; RRID: AB_2800510
anti-fucosyltransferase 8 (rabbit)	Abcam	ab191571; RRID: AB_2800511
anti-beta-1,4-galactosyltransferase 1 (goat)	R&D Systems	AF3609; RRID: AB_2061205
anti-LAMP1 (mouse)	Dr Paul Pryor (University of York)	n/a
anti-GAPDH (mouse)	Applied Biosystems	AM4300
anti-MAN1	Thermo Fisher	PA5-62757; RRID: AB_2643716
anti-endo-mannosidase	Abnova	PAB20528; RRID: AB_10963277
Chemicals, Peptides, and Recombinant Proteins		
Swainsonine	Cambridge Bioscience	16860-5mg-CAY
Deposited Data		
Glcyan profiling, imaging, and western blot data	Research Data York	https://doi.org/10.15124/97b5c373-e7ee-4975-b82f-7147be59d197
Proteomics data for Table S2 and Figure S6	PRIDE	Accession number: PXD013211<br>DOI: 10.6019/PXD013211
Experimental Models: Cell Lines		
HeLa cell line	Dr Daniel Ungar (University of York) (Abdul Rahman et al., 2014)	n/a
Cog4 knockdown HeLa cell line	Dr Daniel Ungar (University of York) (Abdul Rahman et al., 2014)	n/a
Human embryonic kidney 293T (HEK293T) cell line	Professor Vladimir Lupashin (University of Arkansas for Medical Sciences) (Bailey Blackburn et al., 2016)	n/a
Cog4 knockout HEK293T cell line	Professor Vladimir Lupashin (University of Arkansas for Medical Sciences) (Bailey Blackburn et al., 2016)	n/a
hTERT-mesenchymal stromal cell line Y101	Professor Paul Genever (University of York) (James et al., 2015)	n/a
hTERT-mesenchymal stromal cell line Y201	Professor Paul Genever (University of York) (James et al., 2015)	n/a
Recombinant DNA		
Galactosylatransferase 4 -YFP	Dr Daniel Ungar (University of York) (Cottam et al., 2014)	n/a
Software and Algorithms		
Data Analysis 4.2	Brucker Daltonics	n/a
Xcalibur 4.0	Thermo	n/a
Mascot 2.6.1	Matrix Science	n/a
Progenesis QI	Non Linear Dynamics	n/a
ABC algorithm	This work	n/a
SSA	This work	n/a
Xfect transfection reagent	Takara	631317

CONTACT FOR REAGENT AND RESOURCE SHARING

Further information and requests for resources and reagents should be directed to and will be fulfilled by the Lead Contact, Daniel Ungar (dani.ungar@york.ac.uk).

EXPERIMENTAL MODEL AND SUBJECT DETAILS

Cog4KD HeLa cells were generated by the Ungar group by stable transfection of a Cog4 shRNA plasmid (Abdul Rahman et al., 2014). HEK293T cells and Cog4KO HEK293T cells were a kind gift from Vladimir Lupashin (Bailey Blackburn et al., 2016). Y101 MSCs were a kind gift from Paul Genever (James et al., 2015).

METHOD DETAILS

Cell culture and osteogenic differentiation

Cells were grown in Dulbecco's modified essential medium (DMEM) (Life Technologies) in the presence of glutamax, penicillin/streptomycin and 10% fetal bovine serum (basal medium). Cells were incubated and grown at 37°C with 5% CO_2. Cells were passaged when reaching 90% confluency by resuspension (HEK293T) or trypsinization (HeLa cells and MSCs).

For osteogenic differentiation, 80,000 MSCs were seeded in wells of a 24-well dish. Following overnight incubation, the medium was replaced with osteogenic medium (basal medium supplemented with L-ascorbic acid-2-phosphate (50 μg/mL), β-glycerophosphate (5 mM) and dexamethasone (10 nM)). Cells were grown for 21 days with medium changes every 3-4 days with osteogenic medium. For the conditions requiring treatment with lactose (100 mM) or sucrose (100 mM) medium was changed every day for the first 7 days of osteogenesis and from then on, the cells were grown in osteogenic medium with medium changes every 3-4 days up to day 21.

Modeling framework

Linear notation

In order to perform string substitutions *in silico*, the complex structures of *N*-glycans needed to be represented in a form of linear notation. Such an approach has been taken in previous work modeling *N*-glycosylation (Krambeck and Betenbaugh, 2005). Taking the oligomannose glycan $Man_5GlcNAc_2$ and the complex glycan $NeuAc_1Gal_2GlcNAc_2Man_3GlcNAc_2$ as examples, our linear notation would represent these glycan as:

GlcNAc4.1GlcNAc4.1Man(3.1Man:)_m6.1Man(3.1Man:)_m6.1Man:@
GlcNAc4.1GlcNAc4.1Man(3.1Man2.1GlcNAc4.1Gal:)_m6.1Man2.1GlcNAc4.1Gal6.2Sia:@

In this example the linkage between residues is denoted by the numbers in a conventional manner. Residues enclosed in brackets represent separate branches within the *N*-glycan. The underscore and lowercase letters represent the continuation from the previous residue not enclosed within the brackets. The "": represents the termination of the branch and the "@" denotes the end of the *N*-glycan string. We emphasize that this notation was adopted for computational reasons and should not be seen as an attempt to create alternative glycan nomenclature or ontology.

Stochastic simulation algorithm

Enzymes necessary for the biosynthesis of the observed *N*-glycans were used to simulate the glycan profile, in addition to the proportion of $GlcNAc_2Man_8$, $GlcNAc_2Man_9$, $GlcNAc_2Man_9Glc$ entering the Golgi apparatus and the time spent in each cisterna. For the HeLa cell modeling, this equated to 11 enzymes in three cisternae as the effective rate of each enzyme in each cisterna was represented by a separate parameter. Enzyme competition was implicitly simulated but glycan substrate competition was not, as the processing of the precursor glycans occurred one at a time but with all enzymes present. This was repeated for 10,000 glycans in order to generate a simulated glycan profile. Each glycan is represented using a form of linear notation and reactions are represented by string substitution.

Glycan profiles were simulated using an SSA (Figure 1B) based on the Gillespie, or Gillespie-Doeb, algorithm (Doob, 1945; Gillespie, 1976). For two competing enzymes (E_1 and E_2) the total rate is the sum of the two individual rate equations. A brief summary of this methodology for a two-reaction system is as follows. For two reactions

$$A + E_1 \xrightarrow{k_1} B + E_1$$

$$A + E_2 \xrightarrow{k_2} C + E_2,$$

we can calculate a propensity, r_i for each reaction, defined by

$$r_1 = k_1[A][E_1]$$

$$r_2 = k_2[A][E_2],$$

As well as a total propensity, R,

$$R = k_1[A][E_1] + k_2[A][E_2].$$

An event in the time period t to t+δt is determined by drawing randomly from an exponential distribution with the total rate as its mean. The probability *P* of this event being a given reaction occurring is then calculated by computing the probabilities:

$$p_i = \frac{r_i}{R},$$

with

$$p_1 + p_2 = 1.$$

The time interval δt is then drawn from an exponential distribution.

Approximate Bayesian computation without likelihood (*cf.* Figure S1)

To initially parameterise the model for Bayesian fitting of the HeLa cell glycan profile we had to construct prior distributions for each rate parameter. The total effective enzymatic rate was divided over the different cisternae, based on the enzymes' proposed localization e.g., 80% of the effective enzymatic rate of SiaT was placed in the third cisterna. 80% of the input glycan was $GlcNAc_2Man_8$ and the time in each cisterna was 10 minutes. The shapes of the prior distributions for each enzyme parameter were constructed as log-normal distributions if the enzyme was predominantly in that cisterna or exponential decay if not. Values from the prior distributions were used to generate a simulated glycan profile using the methodology described above. The similarity between the simulated and observed glycan profiles was quantified through a scoring system. The scoring system is defined by:

$$score = \sum_{i=1}^{n} (sem_i - (|obs_i - sim_i|))^2$$

If:

$$|obs_i - sim_i| < sem_i$$

$$score = 0$$

Where sem_i is the standard error of the mean for the observed glycan *i*, obs_i and sim_i are the relative abundances of glycan *i* in the observed and simulated dataset respectively.

The prior distributions were sampled and the score reduced by 10% if the acceptance rate of parameter values was greater than 7% (Sherlock et al., 2015). The score was lowered until it reached a user defined threshold at which point the algorithm sampled in that region until 10,000 parameter values for each variable had been accepted. Accepted parameter values were used to construct the posterior distribution. This methodology is equivalent to the acceptance barriers in, for example (Toni and Stumpf, 2010).

Following fitting, the mean parameter values from < 10 individual fitting runs were used to generate simulated glycan profiles. Glycans produced at a relative abundance under 0.1% were removed. Although glycan isoforms can be distinguished within the model, isoforms were collated together to generate one value. Computational simulations were run in parallel on the York Advanced Research Computing Cluster. To assess convergence of multiple Markov chains, the Gelman-Rubin R-Statistic was used (Gelman and Rubin, 1992) and the chains visually assessed to confirm they did accumulate close to the bounds of the prior distribution. The Gelman-Rubin statistic compares the in-chain variance with the between-chain variance providing assurance that the posterior distribution has been explored to a satisfactory extent. For model development with the HeLa and HEK293T cell line data, the oligomannose distribution was modeled initially in isolation before combining the fitting with complex and hybrid glycans.

Once a threshold was reached, the posterior distributions were assessed against the prior distributions. We are conscious that in our method we have no means to access biological rate constants directly. Hypothetically, this could be achieved by assessing mRNA expression, however, this does not necessarily give a reliable estimation of protein levels. In addition, while enzymatic procession rates are based on reported measured values, these are typically from *in vitro*, not *in vivo* measurements. To avoid large amounts of time evaluating space in the tails of prior distributions in a high dimensional parameter space, we permitted ourselves to move prior distributions between fitting runs where there was reasonable biological and numerical evidence to do so. A Mann-Whitney U test was used to assign significance in means between the prior and posterior distributions for each parameter. Only if the change in means was found to be significant was the prior distribution for that parameter altered, and the rejection algorithm run again to a lower threshold. This is a statement of our lack of certainty regarding the composition of the prior distribution due to the absence of direct measurements for the desired rates, and our need to avoid prior distributions that are flat, or nearly flat, due to that large parameter space we are operating in.

Confocal Microscopy

WT and Cog4KO HEK293T cells were transfected with GalT-YFP (Cottam et al., 2014) using Xfect (Clontech) following the manufacturer's instructions. 24 hours post-transfection 60,000 cells were seeded on poly-lysine-treated glass coverslips in a 24-well dish. 45 hours after transfection cells were treated with nocodazole (5 μM) for 3 hours. After nocodazole treatment the cells were washed with PBS and then fixed in 4% paraformaldehyde (Thermo Scientific) for 15 minutes. Cells were then washed with PBS, then glycine (20 mM glycine in PBS) and then incubated in blocking solution (2% BSA, 0.1% saponin, 20 mM glycine in PBS) for 30 minutes. Cells

were stained as indicated with anti-GM130-Alexa647 (1/400, BD Biosciences), anti-MAN1 (1/100, Thermo), anti-endo-mannosidase (1/100, Abnova) for 1 hour before being washed in blocking solution and mounted (Immunomount, Genetex) onto slides. Slides were imaged on a Zeiss LSM 880 operating in Airyscan mode with a 63 × oil objective. Pearson's correlation coefficients were calculated for each individual Golgi stack and averaged for each cell analyzed. Images were analyzed using ImageJ.

Alizarin red staining

Cells were first gently washed with PBS before fixation in 4% formaldehyde (Kautex) for 20 minutes. Formaldehyde was then removed with three PBS washes prior to incubation with Alizarin Red S (40 mM, pH 4.2) for 20 minutes at room temperature. Cells were then washed three times with PBS followed by excessive washes with tap water. Images were acquired on a stereo microscope fitted with an Axiocam MrC5 (Zeiss). After drying, the alizarin red stain was eluted into 200 μL 10% cetylpyridinium chloride for 1 hour while shaking at room temperature. The supernatant was transferred into a 96-well plate and the absorbance read at 570 nm with a microplate spectrophotometer (Multiskan G0, Thermo).

Western blotting

Cells from a 6-well plate at ~90% confluency were gently washed twice with PBS before 200 μL of SDS-PAGE buffer (5% glycerol, 50 mM Tris pH 6.8, 50 mM DTT, 1% SDS, 0.74 mM bromophenol blue) was added to each well and incubated at 20°C for 5 minutes. Samples were then transferred into a microcentrifuge tube and heated at 95°C for 5 minutes before storage at −20°C.

Aliquots of lysate samples (10-20 μL) were loaded into an SDS-PAGE gel and run at 100 V through the stacking gel and then 180 V through the separating gel until adequate separation of the pre-stained ladder (All Blue, Bio-Rad) had occurred. Proteins were transferred onto a polyvinylidene fluoride membrane (Immun-blot, Bio-Rad) by semi-dry transfer. Following transfer, the membrane was blocked in 5% milk powder in PBS with 0.05% tween-20 for 1 hour. Primary antibodies were incubated with the membrane overnight in blocking buffer at 4°C. Primary antibodies used in this study were anti-LAMP1 (1/100, gift from Paul Pryor, University of York), anti-GalT (1/500, R&D Systems, AF3609), anti-MGAT1 (1/500, Abcam, ab180578), anti-FUT8 (1/1000, Abcam, ab191571) and anti-GAPDH (1/500,000, Applied Biosystems). The membrane was washed four times with the blocking buffer before incubation at 20°C for 1 hour with HRP conjugated secondary antibody diluted in blocking buffer. The membrane was then washed a further three times with the blocking buffer and then two times with PBS containing 0.05% tween-20. The membrane was imaged on a Syngene GeneGenius system following exposure to BM Chemiluminescence Western Blotting Substrate (Roche) and densitometry carried out with ImageJ. Glycosylation enzyme bands were normalized to the levels of GAPDH. The WT and Cog4KO band intensities were quantified from the same membrane.

N-glycan release

All glycan profiling data have been previously published with the exception of those from the swainsonine-treated HEK293T cells. The use of MALDI-MS for the analysis of permethylated glycans as applied here has been demonstrated to provide reliable relative quantification (Wada et al., 2007). HEK293T cells were treated with swainsonine (Cayman Chemicals) at 10 μg/mL for 48 hours prior to harvesting for glycan analysis. At ~90% confluency cells were washed five times with PBS and harvested by scraping into a microcentrifuge tube and then centrifuged at 14000 g for 5 minutes at 4°C. The supernatant was removed and lysis buffer (4% (w/v) SDS, 100 mM Tris/HCl pH 7.6, 0.1 M DTT) was added at ten times the pellet volume. The sample was incubated at 97°C for 5 minutes and centrifuged at 14000 g for 10 minutes. The supernatant was then stored at −80°C.

The thawed supernatant was diluted tenfold in urea solution (8 M urea in 100 mM Tris/HCl pH 8.5) and centrifuged in an ultrafiltration tube (Amicon Ultra-0.5, Ultracel-30 membrane, nominal mass cutoff 30 kDa, Millipore) in 400 μL increments at 15000 g for 10 minutes each until the complete sample passed through the filter. The membrane filter was washed with the urea solution three times, before the addition of iodoacetamide solution (40 mM in 300 μL urea solution). The sample was incubated in the dark for 15 minutes and then centrifuged at 14000 g for 10 minutes. Following this the sample was washed with urea solution once followed by ammonium bicarbonate (300 μL, 50 mM pH 8) three times. The membrane filter was incubated at 37°C for 16 hours with 8 U of PNGase F in 100 μL ammonium bicarbonate (50 mM) solution. Released glycans were then eluted into a fresh collection tube by centrifugation of the ammonium bicarbonate and an additional spin with 100 μL water (HPLC grade).

Permethylation

The eluted *N*-glycans were transferred to glass tubes and dried in a vacuum concentrator. The sample was twice washed with 100 μL water (HPLC grade) and dried each time. 20 drops of dimethyl-sulfoxide (DMSO) followed by two heaped microspatulas of finely ground sodium hydroxide pellets were then added to the dried glycans. Iodomethane was then added to the sample as follows: 10 drops followed by 10 minute incubation; 10 drops followed by 10 minute incubation; 20 drops followed by 20 minute incubation. Sodium thiosulfate (1 mL, 100 mg/mL) and dichloromethane (1 mL) were then immediately added to the sample. The organic and aqueous phases were mixed by vortexing and then allowed to separate. The aqueous layer was removed and the organic layer was washed with a similar volume of water (HPLC grade) three or four times. The organic layer was then dried in a vacuum concentrator.

Glycan mass spectrometry

Permethylated glycan samples were redissolved in 20 μL of methanol, and the glycan solution was mixed in a 1:1:0.5 ratio with 20 mg/mL 2,5-dihydroxybenzoic acid (DHB) in 70% methanol and sodium nitrate (0.5 M). 2 μL of this mixture was then spotted onto a MALDI target plate and allowed to air dry. Mass spectra were acquired on a 9.4 T SolariX FT-ICR mass spectrometer (Bruker Daltonics) recorded over the *m/z* range 400-4000 in positive ion mode with 500 laser shots. 8 scans were averaged, and the laser power was set between 30 and 60%. Spectra were calibrated externally using Bruker Peptide Mix II.

Crude membrane proteomics

Cells were grown to near confluency in a 10 cm tissue culture dish before being harvested by centrifugation at 800 × g for three minutes at room temperature. The resulting pellet was then washed twice with PBS and then twice in ice cold permeabilization buffer (20 mM HEPES pH 7.4, 150 mM potassium acetate, 5 mM magnesium acetate, 5 mM DTT), before incubation in digitonin solution (0.1% digitonin in permeabilization buffer) at 4°C with rotation for 30 minutes. Subsequently the total cell membrane fraction was pelleted by centrifugation at 800 × g, 4°C. The membrane pellet was then resuspended in urea lysis buffer (20 mM HEPES pH 8.0, 9 M urea, 1 mM sodium orthovanadate, 2.5 mM sodium pyrophosphate and 1 mM β-glycerophosphate) and lysed with a sonic probe, before clearing the lysate through centrifugation at 20,000 × g for 15 minutes and transferring the supernatant into a LoBind microcentrifuge tube (Eppendorf).

Proteins were reduced with 5 mM dithiothreitol and incubation at 55°C for 30 mins before alkylating with 15 mM iodoacetamide for 30 min at room temperature. Solutions were diluted to 2 M urea with aqueous 50 mM ammonium bicarbonate before digesting with the addition of 1 mg of trypsin/Lys-C protease mixture (Promega) and incubation at 37°C. Digestion was stopped after 16 h with addition of trifluoroacetic acid (TFA) to 0.1% (v:v). Peptides were desalted using Strata 50 mg C_{18} cartridges (Phenomenex). Cartridges were prepared by passing through 3 mL acetonitrile, 2 mL aqueous 80% (v:v) acetonitrile 0.1% (v:v) TFA and 2 mL aqueous 0.1% (v:v) TFA. Peptides were loaded and cartridges washed with 2 × 0.25 mL aqueous 0.1% (v:v) TFA. Peptides were eluted with aqueous 80% (v:v) acetonitrile 0.1% (v:v) TFA before drying in a vacuum concentrator and reconstituting in aqueous 0.1% (v:v) TFA. A common sample pool was created by taking equal aliquots of all samples.

Samples were loaded onto an UltiMate 3000 RSLCnano HPLC system (Thermo) equipped with a PepMap 100 Å C18, 5 μm trap column (300 μm x 5 mm Thermo) and a PepMap, 2 μm, 100 Å, C18 EasyNano nanocapillary column (75 μm x 500 mm, Thermo). The trap wash solvent was aqueous 0.05% (v:v) trifluoroacetic acid and the trapping flow rate was 15 μL/min. The trap was washed for 3 min before switching flow to the capillary column. Separation used gradient elution of two solvents: solvent A, aqueous 1% (v:v) formic acid; solvent B, aqueous 80% (v:v) acetonitrile containing 1% (v:v) formic acid. The flow rate for the capillary column was 300 nL/min and the column temperature was 40°C. The linear multi-step gradient profile was: 3%–10% B over 8 mins, 10%–35% B over 115 mins, 35%–65% B over 30 mins, 65%–99% B over 7 mins and then proceeded to wash with 99% solvent B for 4 min. The column was returned to initial conditions and re-equilibrated for 15 min before subsequent injections.

The nanoLC system was interfaced with an Orbitrap Fusion hybrid mass spectrometer (Thermo) with an EasyNano ionisation source (Thermo). Positive ESI-MS and MS2 spectra were acquired using Xcalibur software (version 4.0, Thermo). Instrument source settings were: ion spray voltage, 1,900 V; sweep gas, 0 Arb; ion transfer tube temperature; 275°C. MS1 spectra were acquired in the Orbitrap with: 120,000 resolution, scan range: m/z 375-1,500; AGC target, 4e5; max fill time, 100 ms. Data-dependent acquisition was performed in top speed mode using a fixed 1 s cycle, selecting the most intense precursors with charge states 2-5. Easy-IC was used for internal calibration. Dynamic exclusion was performed for 50 s post precursor selection and a minimum threshold for fragmentation was set at 5e3. MS2 spectra were acquired in the linear ion trap with: scan rate, turbo; quadrupole isolation, 1.6 *m/z*; activation type, HCD; activation energy: 32%; AGC target, 5e3; first mass, 110 *m/z*; max fill time, 100 ms. Acquisitions were arranged by Xcalibur to inject ions for all available parallelizable time.

Peak lists in .raw format were imported into Progenesis QI (Version 2.2., Waters) and LC-MS runs aligned to the common sample pool. Precursor ion intensities were normalized against total intensity for each acquisition. A combined peak list was exported in .mgf format for database searching against the human subset of the UniProt database. Mascot Daemon (version 2.6.1, Matrix Science) was used to submit the search to a locally-running copy of the Mascot program (Matrix Science Ltd., version 2.6.1). Search criteria specified: Enzyme, trypsin; Max missed cleavages, 2; Fixed modifications, carbamidomethyl (C); Variable modifications, oxidation (M); Peptide tolerance, 3 ppm; MS/MS tolerance, 0.5 Da; Instrument, ESI-TRAP. Peptide identifications were passed through the percolator algorithm to achieve a 1% false discovery rate and individual match filtered to require a minimum expect score of 0.05. The Mascot .XML result file was imported into Progenesis QI and peptide identifications associated with precursor peak areas. Normalization was performed between acquisitions against total precursor intensities for a subset of known ER proteins. Relative protein abundance was calculated using precursor ion areas from non-conflicting unique peptides. Statistical testing was performed in Progenesis QI and ANOVA-derived p values were converted to multiple test-corrected q-values using the Hochberg and Benjamini approach.

Beta-hexosaminidase assay

Cells were seeded in a 24-well tissue culture plate, harvested in PBS, pelleted with centrifugation at 800 × g, and resuspended in citrate buffer (100 μL, 40 mM sodium citrate, 60 mM citric acid, 0.15% Triton X-100). The lysate (25 μL) was added to the bottom

of a clear plastic tube, followed by 100 μL substrate solution (100 mM citrate buffer, pH 5.0, 0.5 mM 4-methylumbelliferyl-2-acetamido-2-deoxy-beta-D-glucopyranoside, 0.27 M sucrose). After exactly three minutes the reaction was stopped with the addition of 1 mL 1 M sodium carbonate. Fluorometric quantification was achieved by exciting at 360 nm wavelength and reading emission at 445 nm.

QUANTIFICATION AND STATISTICAL ANALYSIS

Student's t tests were used to assess significance between band intensities from western blotting and for comparison of Pearson's correlation coefficient. Band intensities and Pearson's correlation coefficients are shown as mean ± standard deviation. Pearson's correlation coefficients were calculated using ImageJ. Mann Whitney-U tests were used to determine statistical significance between simulated parameters from independent fitting runs. *P value*s are denoted by: ns, $p > 0.05$; *, $p < 0.05$; **, $p < 0.01$; ***, $p < 0.001$. Further details of statistical tests used can be found in the relevant figure legends.

DATA AND SOFTWARE AVAILABILITY

The software used for modeling and fitting glycan profiles can be requested by emailing Daniel Ungar (dani.ungar@york.ac.uk) or A. Jamie Wood (jamie.wood@york.ac.uk).

The glycan profiling, imaging and western blot data have been deposited at https://doi.org/10.15124/97b5c373-e7ee-4975-b82f-7147be59d197

The proteomics data used for Table S3 and Figure S6 have been deposited at https://doi.org/10.6019/PXD013211. The accession number is PRIDE: PXD013211.

Cell Reports, Volume *27*

Supplemental Information

Modeling Glycan Processing Reveals Golgi-Enzyme Homeostasis upon Trafficking Defects and Cellular Differentiation

Peter Fisher, Hannah Spencer, Jane Thomas-Oates, A. Jamie Wood, and Daniel Ungar

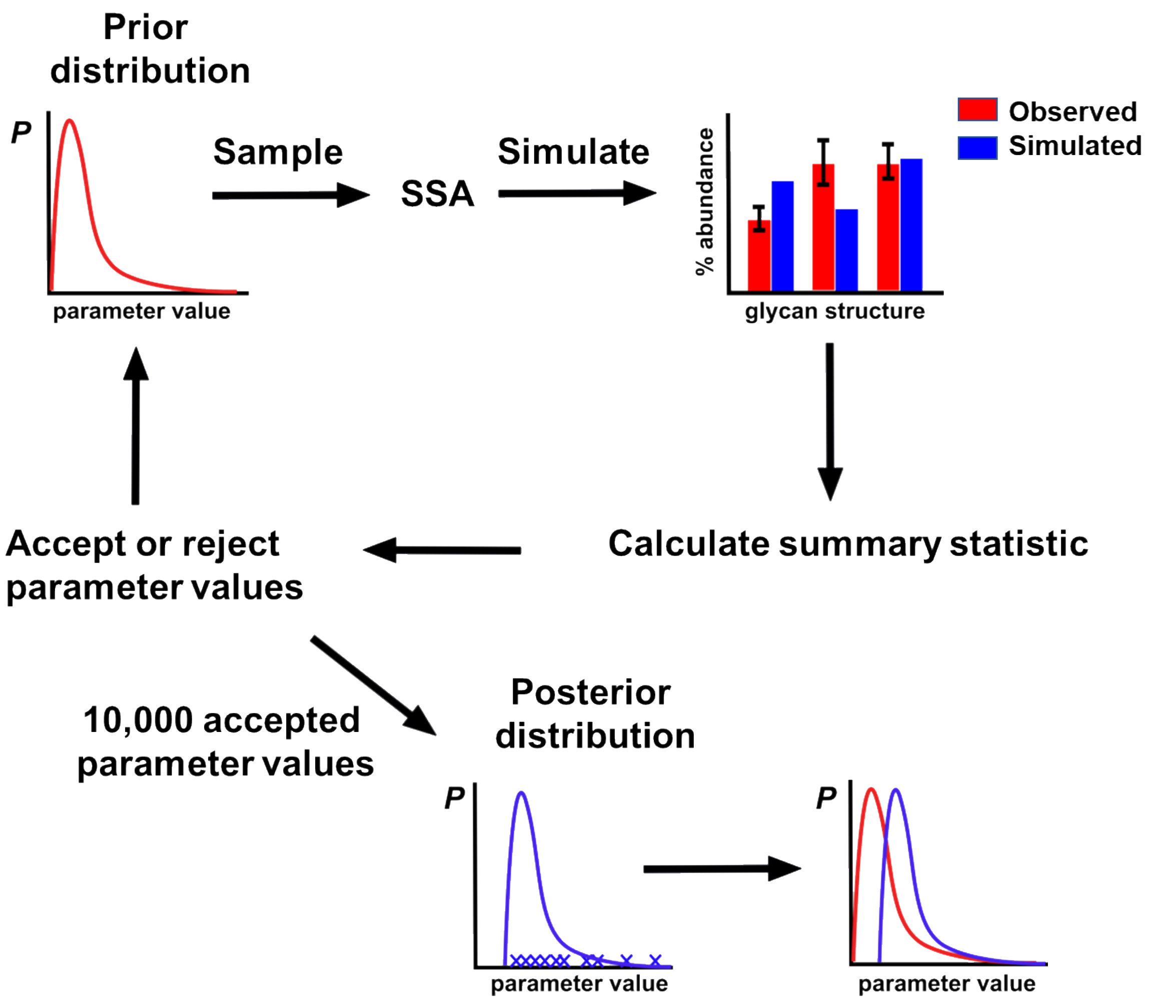

Figure S1. related to figure 1 | Pictorial representation of the Approximate Bayesian Computation fitting procedure.

A parameter value is sampled from prior distribution and used to simulate a glycan profile. The simulated glycan profile is compared to the observed glycan profile and a summary statistic calculated. The parameter value is accepted if the similarity between the simulated and observed glycan profiles is below a user defined threshold, otherwise the parameter value is rejected. This process is repeated until the lowest possible score is achieved at the optimal acceptance ratio, then 10,000 values are sampled in order to construct a posterior distribution.

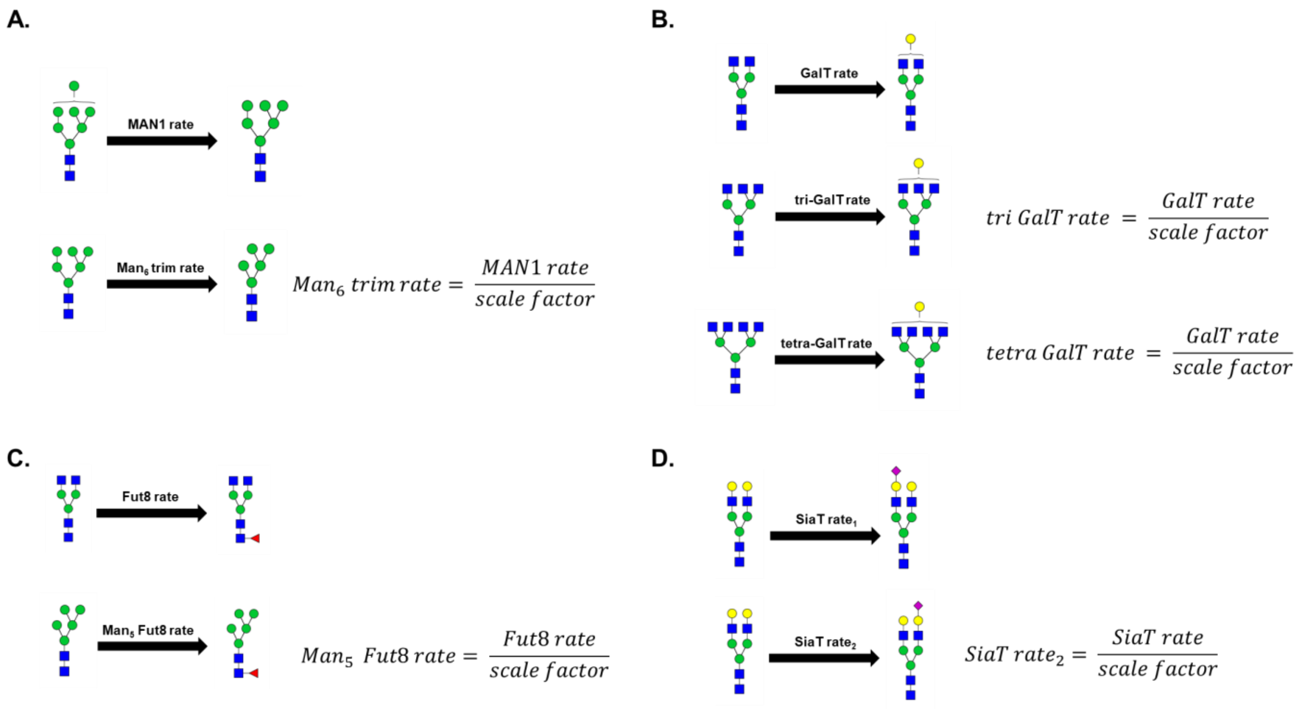

Figure S2. related to figure 2 | Graphical representation of the scale-factors used to simulate substrate specific activities.

(A) For MAN1 to convert $GlcNAc_2Man_6$, (B) For GalT to convert bi-, tri- and tetra-antennary glycans, (C) For FUT8 to convert $GlcNAc_2Man_5$, and (D) For SiaT to add sialic acid to different antennae.

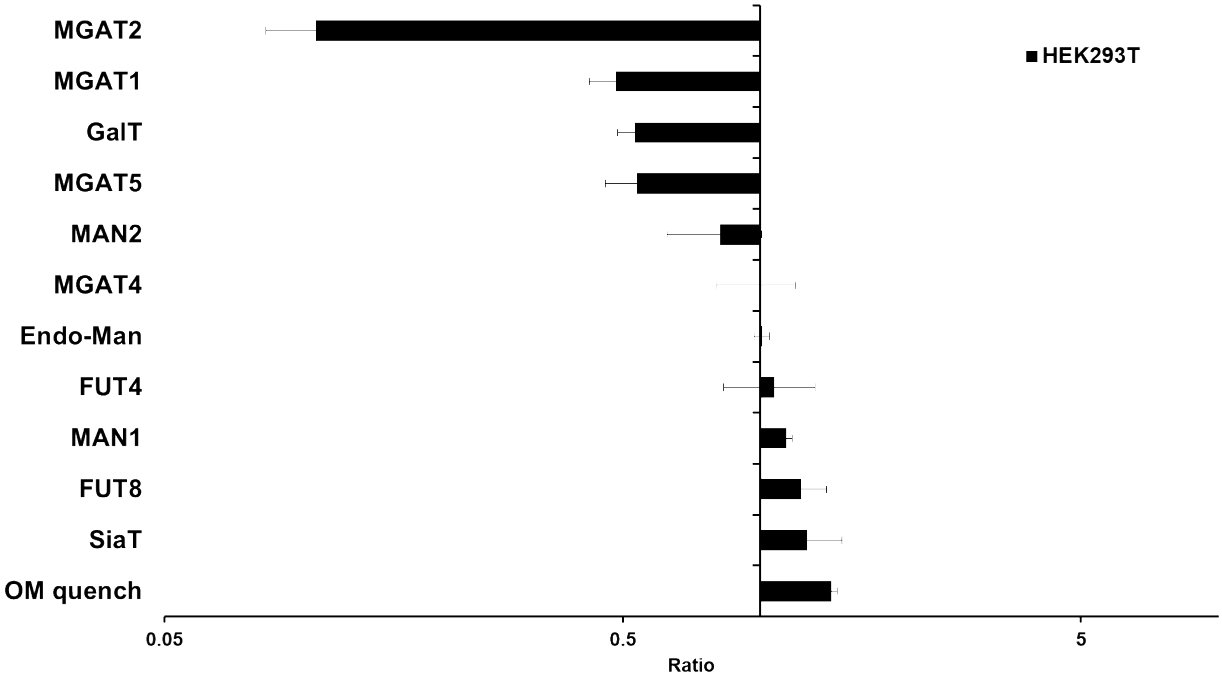

Figure S3. related to figure 4 | Predicted total activity changes between WT HEK293T and Cog4KO HEK293T cells.

This figure shows the graph as displayed in figure 4C, but with MGAT2 included. The reason to exclude MGAT2 from figure 4C is as follows:

Fitting of the WT profile was insensitive to MGAT2 alterations due to the very high activity obtained from the literature during initial parameterization. However, fitting of the Cog4KO profile was sensitive to this parameter. MGAT2 activities were therefore manually reduced in all cisternae prior to Cog4KO fitting, resulting in this enzyme displaying the largest change in activity in this figure. However, the low MGAT2 activities resulting from fitting to the Cog4KO profile did not change the simulated WT glycan profile at all. This implies that the change of this parameter upon Cog4KO is not a biologically meaningful result, but purely due to it being undefined in the WT case.

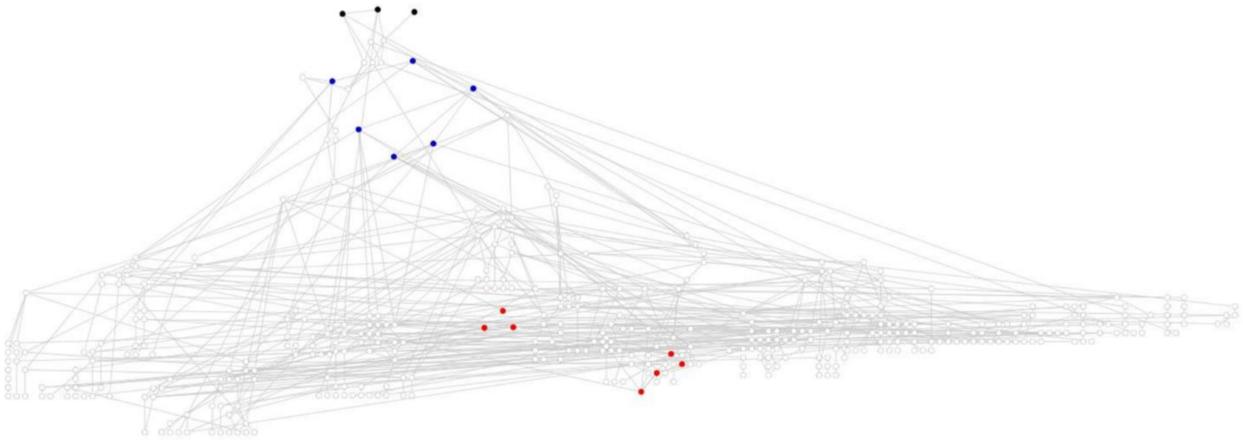

Figure S4. related to figure 5 | Flux map for the model of Cog4KO HEK293T cells.

(A) Total flux map for all enzymatic glycan processing reactions for 10,000 input glycans occurring during the simulation of the Cog4KO HEK293T glycan profile. Blue dots represent the substrates of the six reactions with the highest fluxes of all FUT8 catalysed reactions. Red dots represent the most abundant fucosylated glycans in the observed glycan profile of these cells. Black dots represent the three precursor ER glycans.

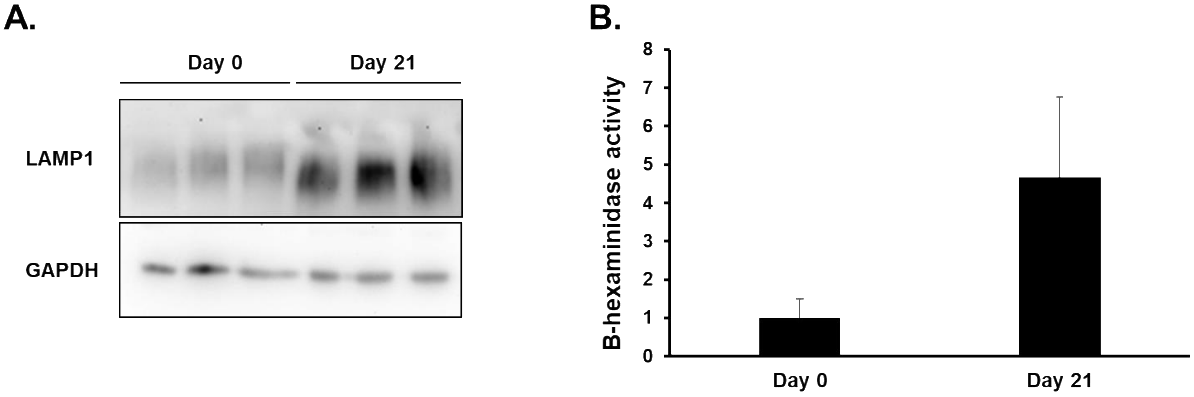

Figure S5. related to figure 6 | Lysosomal content increases upon osteogenesis.

(A) Western blot of LAMP1 in day 0 MSCs and Day 21 osteoblasts. (B) Beta-hexosaminidase activity of day 0 MSCs (n =3) and Day 21 osteoblasts (n = 2). Error bars are standard deviation.

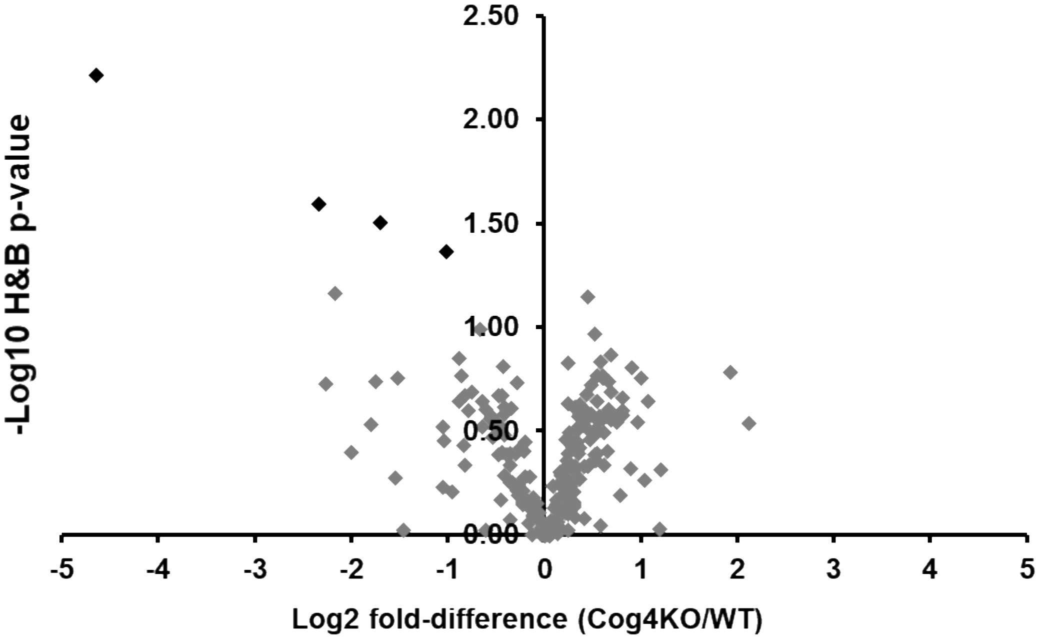

Figure S6. related to figure 4 | Abundance of membrane proteins in WT and Cog4KO HEK293T cells.

Volcano plot representing the ratio of abundance for transmembrane proteins (not including Golgi associated proteins) in WT vs Cog4KO HEK293T cells determined by mass spectrometry. The four black points represent the only proteins which show a significant difference in the Cog4KO HEK293T cells when compared with the WT. Please refer to supplementary table 3 for raw data.

Enzyme Name	Enzyme Target	Enzyme Result
**MANI (1)**	1Man2.1Man:	1Man:
**MANI (2)**	GlcNAc4.1GlcNAc4.1Man(3.1ManSS2.1Man:)_m6.1Man(3.1Man:)_m6.1Man:	GlcNAc4.1GlcNAc4.1Man(3.1Man:)_m6.1Man(3.1Man:)_m6.1Man:
**Endo-mannosidase**	GlcNAc4.1GlcNAc4.1Man(3.1ManSS2.1Man2.1Man3.1Gluc:)_m6.1Man(3.1Man2.1ManGG:)_m6.1Man2.1ManGG:	GlcNAc4.1GlcNAc4.1Man(3.1ManSS2.1Man:)_m6.1Man(3.1Man2.1Man:)_m6.1Man2.1Man:
**MAN2**	Man(3.1Man2.1GlcNAc:)_m6.1Man(3.1Man:)_m6.1Man:	Man(3.1Man2.1GlcNAc:)_m6.1Man:
**MGAT 1**	4.1Man(3.1Man:)_m6.1Man(3.1Man:)_m6.1Man:	4.1Man(3.1Man2.1GlcNAc:)_m6.1Man(3.1Man:)_m6.1Man:
**MGAT 2 (1)**	GlcNAc:)_m6.1Man:	GlcNAc:)_m6.1Man2.1GlcNAc:
**MGAT 2 (2)**	GlcNAc4.1Gal:)_m6.1Man:	GlcNAc4.1Gal:)_m6.1Man2.1GlcNAc:
**MGAT 2 (3)**	GlcNAc4.1Gal6.2Sia:)_m6.1Man:	GlcNAc4.1Gal6.2Sia:)_m6.1Man2.1GlcNAc:
**MGAT 4**	3.1Man2.1GlcNAc:	3.1Man(2.1GlcNAc:)_m4.1GlcNAc:
**MGAT 5**	6.1Man2.1GlcNAc:	6.1Man(2.1GlcNAc:)_m6.1GlcNAc:
**FUT8 (1)**	GlcNAc4.1GlcNAc4.1Man(3.1Man2.1GlcNAc:)_m6.1Man	GlcNAc(6.1Fuc:)_g4.1GlcNAc4.1Man(3.1Man2.1GlcNAc:)_m6.1Man
**FUT8 (2)**	GlcNAc4.1GlcNAc4.1Man(3.1Man(2.1GlcNAc:)_m4.1GlcNAc	GlcNAc(6.1Fuc)_g4.1GlcNAc4.1Man(3.1Man(2.1GlcNAc:)_m4.1GlcNAc
**FUT8 (3)**	GlcNAc4.1GlcNAc4.1Man(3.1Man2.1GlcNAc4.1Gal:)_m6.1Man	GlcNAc(6.1Fuc:)_g4.1GlcNAc4.1Man(3.1Man2.1GlcNAc4.1Gal:)_m6.1Man
**FUT8 (4)**	GlcNAc4.1GlcNAc4.1Man(3.1Man2.1GlcNAc4.1Gal6.2Sia:)_m6.1Man	GlcNAc(6.1Fuc:)_g4.1GlcNAc4.1Man(3.1Man2.1GlcNAc4.1Gal6.2Sia:)_m6.1Man
**FUT8 (5)**	GlcNAc4.1GlcNAc4.1Man(3.1Man(2.1GlcNAc4.1Gal:)_m4.1GlcNAc	GlcNAc(6.1Fuc:)_g4.1GlcNAc4.1Man(3.1Man(2.1GlcNAc4.1Gal:)_m4.1GlcNAc
**FUT8 (6)**	GlcNAc4.1GlcNAc4.1Man(3.1Man(2.1GlcNAc4.1Gal6.2Sia:)_m4.1GlcNAc	GlcNAc(6.1Fuc:)_g4.1GlcNAc4.1Man(3.1Man(2.1GlcNAc4.1Gal6.2Sia:)_m4.1GlcNAc
**FUT8 (7)**	GlcNAc4.1GlcNAc4.1Man(3.1Man:)_m6.1Man(3.1Man:)_m6.1Man:	GlcNAc(6.1Fuc:)_g4.1GlcNAc4.1Man(3.1Man:)_m6.1Man(3.1Man:)_m6.1Man:

**FUT8 off rate**	F:	:
**Antenna FUT**	GlcNAc4.1Gal	GlcNAc(3.1Fuc:)_g4.1Gal
**GalT (1)**	2.1GlcNAc:)_m6.1Man:	2.1GlcNAc4.1Gal:)_m6.1Man:
**GalT (2)**	2.1GlcNAc:)_m6.1Man(3.1Man:)_m6.1Man:	2.1GlcNAc4.1Gal:)_m6.1Man(3.1Man:)_m6.1Man:
**GalT (3)**	2.1GlcNAc:)_m6.1Man2.1GlcNAc	2.1GlcNAc4.1Gal:)_m6.1Man2.1GlcNAc
**GalT (4)**	6.1Man2.1GlcNAc:	6.1Man2.1GlcNAc4.1Gal:
**GalT (5)**	2.1GlcNAc:)_m6.1Man(2.1GlcNAc	2.1GlcNAc4.1Gal:)_m6.1Man(2.1GlcNAc
**GalT (6)**	2.1GlcNAc:)_m4.1GlcNAc	2.1GlcNAc4.1Gal:)_m4.1GlcNAc
**GalT (7)**	6.1Man(2.1GlcNAc:	6.1Man(2.1GlcNAc4.1Gal:
**GalT (8)**	4.1GlcNAc:	4.1GlcNAc4.1Gal:
**GalT (9)**	6.1GlcNAc:	6.1GlcNAc4.1Gal:
**SiaT (1)**	3.1Man2.1GlcNAc4.1Gal:	3.1Man2.1GlcNAc4.1Gal6.2Sia:
**SiaT (2)**	3.1Man(2.1GlcNAc4.1Gal:)_m4.1GlcNAc	3.1Man(2.1GlcNAc4.1Gal6.2Sia:)_m4.1GlcNAc
**SiaT (3)**	.1Gal:	.1Gal6.2Sia:
**OM quench**	1Man2.1Man:	1Man2.1ManP:

Table S1. related to figure 1 | Enzymes and enzyme rules used for wild type cell line modelling.

We worked with the assumption that mainly glycans containing one fucose were fucosylated on the core by FUT8. *N*-glycans that were doubly fucosylated were assumed to be fucosylated on the core and antenna. The glycan profiling data used in this work does not distinguish between the different isoenzymes that fucosylated on the antennae hence the annotation antenna (ant) fucosylation, which groups together all the fucosylation that we assume is on antennae rather than in the core.

Parameter	Initial value	Fitted HeLa	Fitted HEK293T	Fitted hTERT MSC
**$Man_6GlcNAc_2$ to $Man_5GlcNAc_2$**	10	6.27	8.87	11.40
**Fucosylation of $Man_5GlcNAc_2$**	20	1.24[a]	2.90[b]	-
**Sialylation of separate branches**	1	-	2.18	1.82
**Galactosylation of bi- vs tri- and tetra-antennary glycans**	1	-	2.81	1.99

Table S2. related to figure 2 | Enzymatic substrate specificities.

Scaling factors included in the modelling, to account for substrate-specific rate differences for selected enzymes. Numbers shown are dividing scale factors; for example, the reaction converting $Man_6GlcNAc_2$ to $Man_5GlcNAc_2$ is 6.27 times slower than other MAN1-catalysed reactions for HeLa cells.

[a,b] The fitted value for the fucosylation of $Man_5GlcNAc_2$ is for the swainsonine-treated condition. See also Figure S2.

Table S3. related to figure 4 | Crude total membrane proteomics.

Comparison of protein levels in WT and COG4KO HEK293T cells**.** Four proteins (highlighted) change significantly between the two cell types but otherwise the overall membrane-proteomes are similar**.** Golgi proteins were removed to account for the fact that the different Golgi morphology in the mutant could potentially cause selective loss of the organelle during digitonin treatment. This is an Excel file linked to the online version of this paper.

Enzyme Name	Effective enzymatic rate (cisterna 1)	Effective enzymatic rate (cisterna 2)	Effective enzymatic rate (cisterna 3)
**MAN1**	0.233496705	0.527139663	0.178111358
**MAN1(2)**	0.037217926	0.084022791	0.028389845
**EndoMAN**	0.029461455	0.003994726	0.004010006
**MAN2**	0.453718821	3.451994737	0.568873221
**MGAT1**	0.01393476	0.304813621	0.009163032
**MGAT2(1)**	1.798471895	12.77035395	1.700470263
**MGAT2(2)**	1.798471895	12.77035395	1.700470263
**MGAT2(3)**	1.798471895	12.77035395	1.700470263
**MGAT4**	0.004194392	0.031407158	0.004211912
**MGAT5**	0.020214963	0.307998276	0.020739824
**FUT8(1)**	0.049015259	0.412099132	0.053306307
**FUT8(2)**	0.049015259	0.412099132	0.053306307
**FUT8(3)**	0.049015259	0.412099132	0.053306307
**FUT8(4)**	0.049015259	0.412099132	0.053306307
**FUT8(5)**	0.049015259	0.412099132	0.053306307
**FUT8(6)**	0.049015259	0.412099132	0.053306307
**FUT8(7)**	0.000147627	0.001514319	0.000146666
**GalT(1)**	0.451799263	0.448824895	3.148618158
**GalT(2)**	0.451799263	0.448824895	3.148618158
**GalT(3)**	0.451799263	0.448824895	3.148618158
**GalT(4)**	0.451799263	0.448824895	3.148618158
**SiaT**	0.006008948	0.006231213	0.127969208
**OM quench**	0.125711779	0.014646036	0.014101402
	0.710849474[1]	0.009048474[2]	5.279178421[3]

Table S4. related to figure 2 | WT HeLa fitted parameters.

Table representing the fitted parameter values for WT HeLa cells. [1]proportion of $Man_8GlcNAc_2$ as input glycan, [2]proportion of $GluMan_9GlcNAc_2$ as input glycan, [3]transit time per cisterna.

Enzyme Name	Effective enzymatic rate (cisterna 1)	Effective enzymatic rate (cisterna 2)	Effective enzymatic rate (cisterna 3)	Effective enzymatic rate (cisterna 4)
**MAN1**	0.194485087	1.7516927	0.22349944	0.022924432
**MAN1(2)**	0.021928699	0.197507902	0.025200142	0.002584789
**EndoMAN**	0.030639145	0.005333927	0.004076729	0.000423947
**MAN2**	0.50131336	2.9467012	1.914476833	0.547513267
**MGAT1**	0.007119538	0.636804333	0.098816213	0.009268861
**MGAT2(1)**	1.796796067	6.5077812	5.896882133	1.7318214
**MGAT2(2)**	1.796796067	6.5077812	5.896882133	1.7318214
**MGAT2(3)**	1.796796067	6.5077812	5.896882133	1.7318214
**MGAT4**	0.004176106	0.02006288	0.019514897	0.004981294
**MGAT5**	0.020047691	0.168627987	0.24528994	0.031690672
**FUT8(1)**	0.049176632	0.177289367	0.071655067	0.04276546
**FUT8(2)**	0.049176632	0.177289367	0.071655067	0.04276546
**FUT8(3)**	0.049176632	0.177289367	0.071655067	0.04276546
**FUT8(4)**	0.049176632	0.177289367	0.071655067	0.04276546
**FUT8(5)**	0.049176632	0.177289367	0.071655067	0.04276546
**FUT8(6)**	0.049176632	0.177289367	0.071655067	0.04276546
**FUT8(7)**	0.002713389	0.009782188	0.003953668	0.002359644
**FUT8OFF**	0.099446587	0.099446587	0.099446587	0.099446587
**Ant FUT**	0.000999622	0.014081887	0.011049582	0.001026963
**GalT(1)**	0.010020635	0.072018267	0.2061617	0.25531468
**GalT(2)**	0.010020635	0.072018267	0.2061617	0.25531468
**GalT(3)**	0.010020635	0.072018267	0.2061617	0.25531468
**GalT(4)**	0.010020635	0.072018267	0.2061617	0.25531468
**GalT(5)**	0.003562637	0.025604659	0.073296684	0.090772046
**GalT(6)**	0.003562637	0.025604659	0.073296684	0.090772046
**GalT(7)**	0.003562637	0.025604659	0.073296684	0.090772046
**GalT(8)**	0.003562637	0.025604659	0.073296684	0.090772046
**GalT(9)**	0.003562637	0.025604659	0.073296684	0.090772046
**SiaT(1)**	0.006000835	0.006713236	0.084122005	0.09841716
**SiaT(2)**	0.006000835	0.006713236	0.084122005	0.09841716
**SiaT(3)**	0.002754859	0.003081907	0.038618664	0.045181272
**OM quench**	0.052862328	0.011772589	0.014043215	0.001410821
	0.1132855[1]	0.030996753[2]	7.2580224[3]	

Table S5. related to figure 2 | WT HEK293T fitted parameters.

Table representing the fitted parameter values for WT HEK293T cells. [1]proportion of $Man_8GlcNAc_2$ as input glycan, [2]proportion of $GlcMan_9GlcNAc_2$ as input glycan, [3]transit time per cisterna.

Enzyme Name	Effective enzymatic rate (cisterna 1)	Effective enzymatic rate (cisterna 2)	Effective enzymatic rate (cisterna 3)	Effective enzymatic rate (cisterna 4)
**MAN1**	0.15	1.757304643	0.154082229	0.024309597
**MAN1(2)**	0.013169447	0.154202283	0.01352061	0.002133151
**EndoMAN**	0.030250348	0.005568547	0.003948526	0.00041535
**MAN2**	0.506190971	4.903260857	2.659008286	0.545422786
**MGAT1**	0.00693554	0.5	0.1	0.009975619
**MGAT2(1)**	0.179587836	0.564087143	0.727762607	0.248050429
**MGAT2(2)**	0.179587836	0.564087143	0.727762607	0.248050429
**MGAT2(3)**	0.179587836	0.564087143	0.727762607	0.248050429
**MGAT4**	0.004190141	0.014352356	0.018409193	0.005405872
**MGAT5**	0.020134409	0.057334779	0.076209429	0.030761723
**FUT8(1)**	0.048188871	0.15043975	0.057443964	0.057784225
**FUT8(2)**	0.048188871	0.15043975	0.057443964	0.057784225
**FUT8(3)**	0.048188871	0.15043975	0.057443964	0.057784225
**FUT8(4)**	0.048188871	0.15043975	0.057443964	0.057784225
**FUT8(5)**	0.048188871	0.15043975	0.057443964	0.057784225
**FUT8(6)**	0.048188871	0.15043975	0.057443964	0.057784225
**FUT8(7)**	0.002654511	0.008287059	0.003164333	0.003183077
**FUT8OFF**	0.082556143	0.082556143	0.082556143	0.082556143
**Ant FUT**	0.000998262	0.013721026	0.0007	0.0007
**GalT(1)**	0.010078676	0.065822614	0.784380357	0.483927857
**GalT(2)**	0.010078676	0.065822614	0.784380357	0.483927857
**GalT(3)**	0.010078676	0.065822614	0.784380357	0.483927857
**GalT(4)**	0.010078676	0.065822614	0.784380357	0.483927857
**GalT(5)**	0.005056857	0.033025725	0.393553645	0.242805127
**GalT(6)**	0.005056857	0.033025725	0.393553645	0.242805127
**GalT(7)**	0.005056857	0.033025725	0.393553645	0.242805127
**GalT(8)**	0.005056857	0.033025725	0.393553645	0.242805127
**GalT(9)**	0.005056857	0.033025725	0.393553645	0.242805127
**SiaT(1)**	0.006077692	0.006680604	0.04679148	0.034442945
**SiaT(2)**	0.006077692	0.006680604	0.04679148	0.034442945
**SiaT(3)**	0.003315519	0.003644421	0.025525814	0.018789408
**OM quench**	0.03	0.01252273	0.013914026	0.001323202
	0.3[1]	0.017735158[2]	10.03370857[3]	

Table S6. related to figure 6 | Y101 hTERT MSC fitted parameters.

Table representing the fitted parameter values for hTERT-MSCs (Y101s). [1]proportion of $Man_8GlcNAc_2$ as input glycan, [2]proportion of $GlcMan_9GlcNAc_2$ as input glycan, [3]transit time per cisterna.

Enzyme Name	Effective enzymatic rate (cisterna 1)	Effective enzymatic rate (cisterna 2)	Effective enzymatic rate (cisterna 3)	Effective enzymatic rate (cisterna 4)
**MAN1**	0.307558695	1.7744673	0.17476228	0.026185174
**MAN1(2)**	0.030061662	0.173441485	0.017081763	0.002559413
**EndoMAN**	0.033662145	0.00588473	0.004029459	0.000402867
**MAN2**	0.5079001	6.093339	3.377208	0.587262
**MGAT1**	0.007114036	0.6225316	0.079531065	0.009841085
**MGAT2(1)**	0.18019222	0.46439071	0.7227887	0.26702385
**MGAT2(2)**	0.18019222	0.46439071	0.7227887	0.26702385
**MGAT2(3)**	0.18019222	0.46439071	0.7227887	0.26702385
**MGAT4**	0.004192536	0.009751601	0.017818555	0.005511443
**MGAT5**	0.019955005	0.029678331	0.04895692	0.032459256
**FUT8(1)**	0.047849834	0.154176175	0.0523304	0.05802216
**FUT8(2)**	0.047849834	0.154176175	0.0523304	0.05802216
**FUT8(3)**	0.047849834	0.154176175	0.0523304	0.05802216
**FUT8(4)**	0.047849834	0.154176175	0.0523304	0.05802216
**FUT8(5)**	0.047849834	0.154176175	0.0523304	0.05802216
**FUT8(6)**	0.047849834	0.154176175	0.0523304	0.05802216
**FUT8(7)**	0.002452617	0.007902537	0.002682275	0.002974015
**FUT8OFF**	0.092797335	0.092797335	0.092797335	0.092797335
**Ant FUT**	0.000995563	0.01234311	0.000676167	0.000579693
**GalT(1)**	0.010083057	0.05723082	1.14757235	0.53553684
**GalT(2)**	0.010083057	0.05723082	1.14757235	0.53553684
**GalT(3)**	0.010083057	0.05723082	1.14757235	0.53553684
**GalT(4)**	0.010083057	0.05723082	1.14757235	0.53553684
**GalT(5)**	0.005173889	0.029366679	0.588850366	0.274798416
**GalT(6)**	0.005173889	0.029366679	0.588850366	0.274798416
**GalT(7)**	0.005173889	0.029366679	0.588850366	0.274798416
**GalT(8)**	0.005173889	0.029366679	0.588850366	0.274798416
**GalT(9)**	0.005173889	0.029366679	0.588850366	0.274798416
**SiaT(1)**	0.006091488	0.007073644	0.041135007	0.03149241
**SiaT(2)**	0.006091488	0.007073644	0.041135007	0.03149241
**SiaT(3)**	0.002920068	0.003390883	0.019718831	0.015096473
**OM quench**	0.013152243	0.012452489	0.014328323	0.001424251
	0.301190675[1]	0.012826073[2]	12.091441[3]	

Table S7. related to figure 6 | Osteoblast fitted parameters.

Table representing the fitted parameter values for osteoblasts derived from hTERT-MSCs (Y101s). [1]proportion of $Man_8GlcNAc_2$ as input glycan, [2]proportion of $GlcMan_9GlcNAc_2$ as input glycan, [3]transit time per cisterna.
